# Systematic assessment of COVID-19 host genetics using whole genome sequencing data

**DOI:** 10.1371/journal.ppat.1012786

**Published:** 2024-12-23

**Authors:** Axel Schmidt, Nicolas Casadei, Fabian Brand, German Demidov, Elaheh Vojgani, Ayda Abolhassani, Rana Aldisi, Guillaume Butler-Laporte, DeCOI host genetics group, T. Madhusankha Alawathurage, Max Augustin, Robert Bals, Carla Bellinghausen, Marc Moritz Berger, Michael Bitzer, Christian Bode, Jannik Boos, Thorsten Brenner, Oliver A. Cornely, Thomas Eggermann, Johanna Erber, Torsten Feldt, Christian Fuchsberger, Julien Gagneur, Siri Göpel, Tobias Haack, Helene Häberle, Frank Hanses, Julia Heggemann, Ute Hehr, Johannes C. Hellmuth, Christian Herr, Anke Hinney, Per Hoffmann, Thomas Illig, Björn-Erik Ole Jensen, Verena Keitel, Sarah Kim-Hellmuth, Philipp Koehler, Ingo Kurth, Anna-Lisa Lanz, Eicke Latz, Clara Lehmann, Tom Luedde, Carlo Maj, Michael Mian, Abigail Miller, Maximilian Muenchhoff, Isabell Pink, Ulrike Protzer, Hana Rohn, Jan Rybniker, Federica Scaggiante, Anna Schaffeldt, Clemens Scherer, Maximilian Schieck, Susanne V. Schmidt, Philipp Schommers, Christoph D. Spinner, Maria J. G. T. Vehreschild, Thirumalaisamy P. Velavan, Sonja Volland, Sibylle Wilfling, Christof Winter, J. Brent Richards, DeCOI, André Heimbach, Kerstin Becker, Stephan Ossowski, Joachim L. Schultze, Peter Nürnberg, Markus M. Nöthen, Susanne Motameny, Michael Nothnagel, Olaf Riess, Eva C. Schulte, Kerstin U. Ludwig

**Affiliations:** 1 Institute of Human Genetics, School of Medicine, University Bonn & University Hospital Bonn, Bonn, Germany; 2 Department of Pediatric Neurology, School of Medicine, University Bonn & University Hospital Bonn, Bonn, Germany; 3 DFG NGS Competence Center Tübingen (NCCT), University of Tübingen, Tübingen, Germany; 4 Institute of Medical Genetics and Applied Genomics, University of Tübingen, Tübingen, Germany; 5 Institute of Genomic Statistics and Bioinformatics, School of Medicine, University Bonn & University Hospital Bonn, Bonn, Germany; 6 Institute for Bioinformatics and Medical Informatics (IBMI), University of Tübingen, Tübingen, Germany; 7 Cologne Center for Genomics (CCG), University of Cologne, Cologne, Germany; 8 Department of Psychiatry and Psychotherapy, School of Medicine, University Bonn & University Hospital Bonn, Bonn, Germany; 9 Lady Davis Institute, Jewish General Hospital, McGill University, Montréal, Québec, Canada; 10 Wellcome Trust Centre for Human Genetics, University of Oxford, Oxford, United Kingdom; 11 Center for Molecular Medicine Cologne (CMMC), University of Cologne, Cologne, Germany; 12 Department I of Internal Medicine, Faculty of Medicine and University Hospital Cologne, University of Cologne, Cologne, Germany; 13 German Center for Infection Research (DZIF), Partner Site Bonn-Cologne, Cologne, Germany; 14 Department of Internal Medicine V, Saarland University, Homburg, Germany; 15 Helmholtz Institute for Pharmaceutical Research Saarland (HIPS), Saarbrücken, Germany; 16 Department of Internal Medicine, Pneumology, University Hospital Frankfurt, Goethe University, Frankfurt am Main, Germany; 17 Department of Anesthesiology and Intensive Care Medicine, University Hospital Essen, University Duisburg-Essen, Essen, Germany; 18 Center for Personalized Medicine, University Hospital Tübingen, Tübingen, Germany; 19 Department of Internal Medicine I, University Hospital Tübingen, Tübingen, Germany; 20 Department of Anesthesiology and Intensive Care Medicine, University Hospital Bonn, Bonn, Germany; 21 Clinical Trials Center Cologne, Faculty of Medicine and University Hospital Cologne, University of Cologne, Cologne, Germany; 22 Institute of Translational Research, Cologne Excellence Cluster on Cellular Stress Responses in Aging-Associated Diseases (CECAD), Faculty of Medicine and University Hospital Cologne, University of Cologne, Cologne, Germany; 23 Institute for Human Genetics and Genomic Medicine, Medical Faculty, RWTH Aachen University, Aachen, Germany; 24 Department of Internal Medicine II, University Hospital rechts der Isar, School of Medicine, Technical University of Munich, Munich, Germany; 25 Department of Gastroenterology, Hepatology and Infectious Diseases, University Hospital Duesseldorf, Medical Faculty, Düsseldorf, Germany; 26 Eurac Research, Institute for Biomedicine, Bolzano, Italy; 27 Computational Health Center, Helmholtz Zentrum München, Neuherberg, Germany; 28 Institute of Human Genetics, School of Medicine, Technical University of Munich, Munich, Germany; 29 School of Computation, Information and Technology, Technical University of Munich, Garching, Germany; 30 German Center for Infection Research (DZIF), Partner Site Tübingen, Tübingen, Germany; 31 Department of Anesthesiology and Intensive Care Medicine, University Hospital Tübingen, Tübingen, Germany; 32 Department for Infection Control and Infectious Diseases, University Hospital Regensburg, Regensburg, Germany; 33 Emergency Department, University Hospital Regensburg, Regensburg, Germany; 34 Center for Human Genetics Regensburg, Regensburg, Germany; 35 COVID-19 Registry of the LMU Munich (CORKUM), University Hospital, LMU Munich, Munich, Germany; 36 Department of Medicine III, University Hospital, LMU Munich, Munich, Germany; 37 Department of Child and Adolescent Psychiatry and Psychotherapy, University Hospital Essen, University of Duisburg-Essen, Essen, Germany; 38 Hannover Unified Biobank, Hannover Medical School, Hannover, Germany; 39 Department of Pediatrics, Dr. von Hauner Children’s Hospital, University Hospital LMU Munich, Munich, Germany; 40 Institute of Translational Genomics, Helmholtz Munich, Neuherberg, Germany; 41 Institute of Innate Immunity, University Hospital Bonn, Bonn, Germany; 42 Center for Human Genetics, Philipps University of Marburg, Marburg, Germany; 43 Service for Innovation, Research and Teaching, (SABES-ASDAA), Bolzano-Bozen, Italy; Teaching Hospital of Paracelsus Medical University; 44 Max von Pettenkofer Institute and Gene Center, Virology, National Reference Center for Retroviruses, LMU Munich, Munich, Germany; 45 Department of Pneumology, Hannover Medical School, Hannover, Germany; 46 German Center for Infection research (DZIF), Partner Site Munich, Munich, Germany; 47 Institute of Virology, Technical University Munich/Helmholtz Munich, Munich, Germany; 48 Department of Infectious Diseases, West German Centre of Infectious Diseases, University Hospital Essen, University Duisburg-Essen, Essen, Germany; 49 Laboratorio di Patologia Clinica di Bressanone, Hospital of Bressanone (SABES-ASDAA), Bressanone-Brixen, Italy; Teaching Hospital of Paracelsus Medical University; 50 Department of Medicine I, University Hospital, LMU Munich, Munich, Germany; 51 Department of Internal Medicine, Infectious Diseases, University Hospital Frankfurt, Goethe University, Frankfurt am Main, Germany; 52 Institute of Tropical Medicine, Universitätsklinikum Tübingen, Tübingen, Germany; 53 Vietnamese-German Center for Medical Research (VG-CARE), Hanoi, Vietnam; 54 Department of Neurology, Bezirksklinikum Regensburg, University of Regensburg, Regensburg, Germany; 55 German Cancer Consortium (DKTK), Partner Site Munich, Munich, Germany; 56 German Cancer Research Center (DKFZ), Heidelberg, Germany; 57 Institute of Clinical Chemistry and Pathobiochemistry, Klinikum Rechts der Isar, School of Medicine, Technical University of Munich, Munich, Germany; 58 TranslaTUM, Center for Translational Cancer Research, Technical University of Munich, Munich, Germany; 59 5 Prime Sciences Inc, Montréal, Québec, Canada; 60 Department of Epidemiology, Biostatistics and Occupational Health, McGill University, Montréal, Québec, Canada; 61 Department of Human Genetics, McGill University, Montréal, Québec, Canada; 62 Department of Twin Research, King’s College London, London, United Kingdom; 63 Infectious Diseases and Immunity in Global Health Program, Research Institute of the McGill University Health Centre, Montréal, Québec, Canada; 64 NGS Core Facility Bonn, University of Bonn, School of Medicine & University Hospital Bonn, Bonn, Germany; 65 West German Genome Center ‐ Cologne, University of Cologne, Cologne, Germany; 66 Genomics and Immunoregulation, Life & Medical Sciences (LIMES) Institute, University of Bonn, Bonn, Germany; 67 PRECISE Platform for Genomics and Epigenomics, Deutsches Zentrum für Neurodegenerative Erkrankungen (DZNE) e.V. and University of Bonn, Bonn, Germany; 68 Systems Medicine, Deutsches Zentrum für Neurodegenerative Erkrankungen (DZNE) e.V., Bonn, Germany; 69 Department of Psychiatry & Psychotherapy, University of Munich, Munich, Germany; 70 Institute of Psychiatric Phenomics and Genomics, University of Munich, Munich, Germany; NIAID: National Institute of Allergy and Infectious Diseases, UNITED STATES OF AMERICA

## Abstract

Courses of SARS-CoV-2 infections are highly variable, ranging from asymptomatic to lethal COVID-19. Though research has shown that host genetic factors contribute to this variability, cohort-based joint analyses of variants from the entire allelic spectrum in individuals with confirmed SARS-CoV-2 infections are still lacking. Here, we present the results of whole genome sequencing in 1,220 mainly vaccine-naïve individuals with confirmed SARS-CoV-2 infection, including 827 hospitalized COVID-19 cases. We observed the presence of autosomal-recessive or likely compound heterozygous monogenic disorders in six individuals, all of which were hospitalized and significantly younger than the rest of the cohort. We did not observe any suggestive causal variants in or around the established risk gene *TLR7*. Burden testing in the largest population subgroup (i.e., Europeans) suggested nominal enrichments of rare variants in coding and non-coding regions of interferon immune response genes in the overall analysis and male subgroup. Case-control analyses of more common variants confirmed associations with previously reported risk loci, with the key locus at 3p21 reaching genome-wide significance. Polygenic scores accurately captured risk in an age-dependent manner. By enabling joint analyses of different types of variation across the entire frequency spectrum, this data will continue to contribute to the elucidation of COVID-19 etiology.

## Author summary

After infection with SARS-CoV-2, symptoms vary widely. On average, individuals who are older, males and those with certain comorbidities tend to be more severely affected by COVID-19. Additionally, genetics of the infected individuals (host genetics) modulate the severity of symptoms, but so far, most studies on COVID-19 host genetics have focused either on common or on rare variants, but not both. In this study, we analyzed genetic variants comprehensively by whole genome sequencing of 1,220 SARS-CoV-2 positive individuals with varying degrees of COVID-19 severity. In our cohort, we replicate several associations between common variants and COVID-19 severity, with a region on chromosome 3 showing the largest effect size. We additionally show that common variants, taken together, can help to predict COVID-19 severity, particularly in individuals younger than 60 years. We also identified six individuals with moderate or severe COVID-19 who had underlying rare genetic diseases, which creates interesting new hypotheses. Finally, we observed an enrichment of rare variants in immune pathways in severe or moderate COVID-19. This study provides comprehensive novel insights into COVID-19 host genetics.

## Introduction

Since late 2019, severe acute respiratory syndrome coronavirus type 2 (SARS-CoV-2) has infected hundreds of millions of people worldwide. SARS-CoV-2 infections are clinically heterogeneous and can remain asymptomatic or become symptomatic, the latter being referred to as Coronavirus Disease 2019 (COVID-19). COVID-19 mainly affects the respiratory tract and can lead to severe pneumonia, but other organ systems may also be affected. Research has shown that the clinical heterogeneity of COVID-19 can be explained in part by demographic factors (e.g., advanced age and male sex [1]), and the presence of predisposing medical conditions [2] or auto-antibodies [3]. In addition, epidemiological data have implicated host genetic factors [4].

Through the work of large global consortia, such as the COVID-19 Host Genetics Initiative (COVID-19 HGI) [5], the analyses of data from biobanks, and individual clinical studies, multiple host genetic loci that contribute to an individual’s risk for severe disease secondary to SARS-CoV-2 infection have now been identified [6]. Specifically, genome-wide association studies (GWAS) have highlighted at least 71 loci at which common variants contribute to infection susceptibility or COVID-19 severity [7–10]. These efforts have been complemented by whole exome sequencing (WES) studies of severely affected individuals, which have led to the identification of rare loss-of-function (LoF) variants in genes involved in the innate immune response [11,12], some of which are known inborn errors of immunity or have subsequently been classified as such [13]. At the time of writing, the COVID-19 risk gene with the most compelling evidence in terms of rare variants is the X-chromosomal toll-like receptor 7 gene (*TLR7*), for which LoF variants were initially detected in two pairs of previously healthy young (aged 21–32 years) brothers with severe to fatal disease [14]. Subsequent candidate gene-, machine learning-, and WES-based rare variant association approaches have generated independent support for the role of *TLR7* in severe COVID-19 in males [15–18], with recent estimates suggesting the presence of a TLR7 deficiency in around 1–2% of male cases [15,19]. Besides *TLR7*, additional candidate genes have been suggested, e.g. 13 genes of the type I interferon (IFN) immunity [11,12,20].

To date, most investigations of host genetic factors in SARS-CoV-2 infections have analyzed either common variants (mainly through genome-wide array-based genotyping followed by imputation) [7,8,10,21–27] or rare variants in protein-coding regions (mostly through WES in either clinical cohorts [11,15,17,18,20,28,29]; or families [14,30,31]). However, these approaches fail to cover a substantial fraction of the total genetic variability (such as rare variants in non-coding regions), and are rarely combined on the same individual genomes, thereby precluding joint analyses of variants along the entire allelic spectrum. These issues can be resolved via whole genome sequencing (WGS). To date, however, WGS has rarely been applied in this field because of its relatively high costs and its full potential in COVID-19 has yet to be explored [22,29].

By building on the German COVID-19 Omics Initiative (DeCOI) [32], we established a national consortium to investigate the host genetics of COVID-19 (S1 Table). WGS data of 1,220 individuals with reported SARS-CoV-2 infection and variable disease outcomes were used to characterize genetic risk factors related to COVID-19 severity. We investigated the presence of: (i) potentially causal rare variants within the *TLR7* locus, including adjacent non-coding regions, and in additional 13 candidate genes; (ii) monogenic conditions that might increase the risk for severe COVID-19; and (iii) immune-relevant gene sets (in both coding and non-coding regions) that are enriched for functionally-relevant rare variation. Furthermore, we investigated the polygenic architecture of severe COVID-19 in age-stratified groups. These analyses comprehensively characterize the joint contribution of variants of the entire allelic spectrum to severe COVID-19.

## Results

### The DeCOI cohort

**Fig 1:**
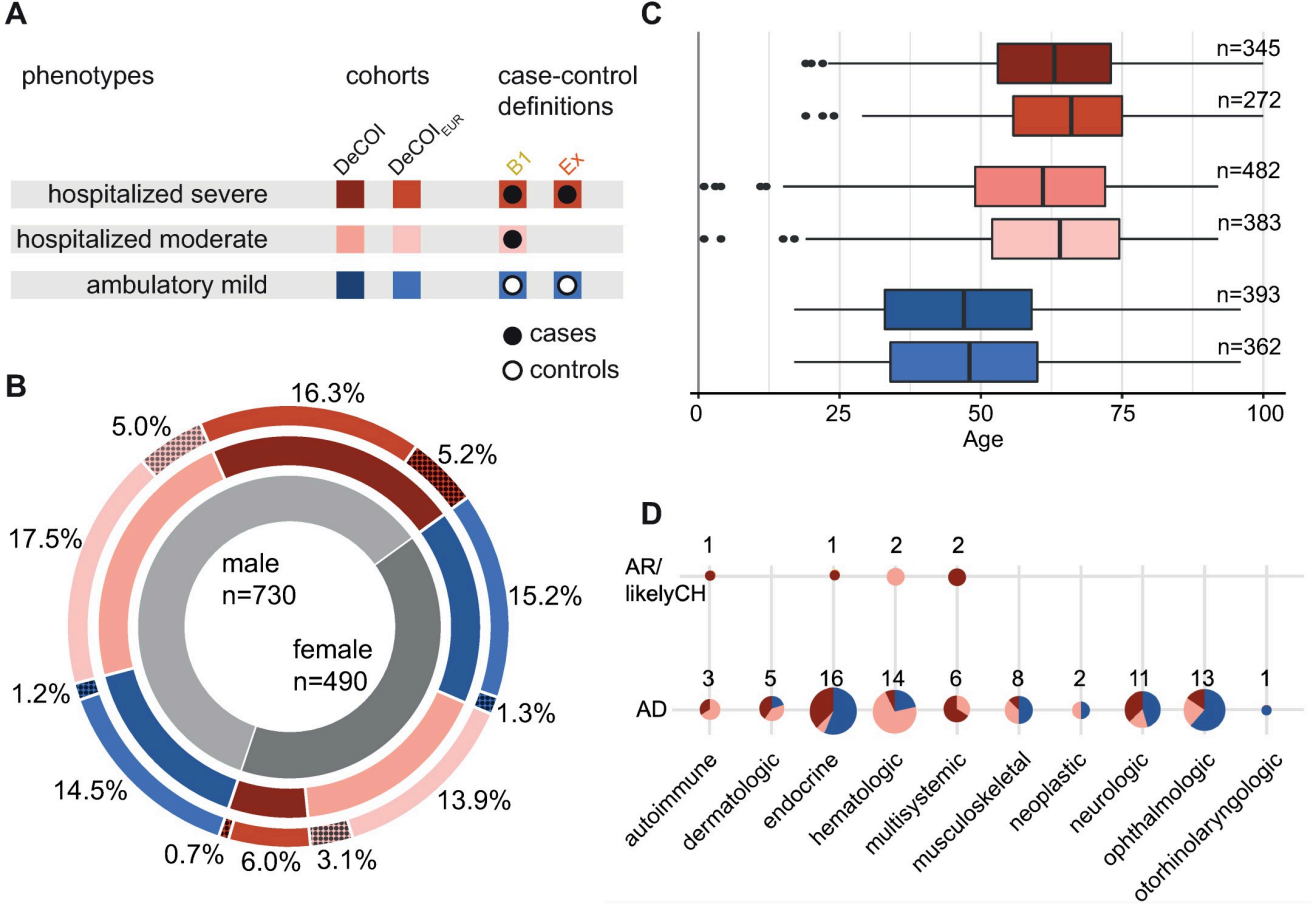
The DeCOI and the DeCOI_EUR_ cohort. (A) Individuals in the DeCOI cohort are classified into three phenotypes based on WHO definition. In addition, the cohort was subsetted to an unrelated cohort of the European population (DeCOI_EUR_) for association analyses. Based on the phenotypes, case-control definitions were established within DeCOI_EUR_. (B) Composition of the DeCOI cohort according to sex (inner circle), phenotype (color coded, middle circle), and population (outer circle). Shaded intervals in the outer circle represent non-European individuals. (C) Age distribution of individuals from the DeCOI cohort (n = 1,220) and the European subcohort (DeCOI_EUR_; n = 1,017), as stratified according to severity (color coded). In both subcohorts, the average age increases with disease course severity. Numbers indicate individuals in the respective group. (D) Phenotype distribution of individuals harboring ClinVar-annotated variants, as grouped according to disorder class. Autosomal recessive patterns of inheritance (AR/likely compound-heterozygous (CH), n = 6 diseases in six individuals) are displayed in the upper panel, and autosomal dominant inheritance patterns (AD, n = 79 diseases in 77 individuals) are displayed in the lower panel.

Following quality control (see Methods), the DeCOI cohort comprised 1,220 individuals from across the entire phenotypic spectrum of SARS-CoV-2 infections (Figs 1A–1C and S1). The average age of the cohort was 56.2 years (range: 1–100 years), and 490 participants were female (40.2%). Based on the available phenotypic information, 393 individuals were classified as having had mild SARS-CoV-2 infections (“ambulatory mild”, World Health Organization ordinal scale for COVID-19 severity (WHO score, [33]) 1–3), 482 individuals were classified as having been hospitalized without the need for high-flow oxygen or mechanical ventilation (“hospitalized moderate”, WHO 4–5), and 345 individuals were classified as having either required at least high-flow oxygen or mechanical ventilation, or having had lethal COVID-19 (“hospitalized severe”, WHO 6–10). Consistent with available epidemiological evidence, both the average age and the proportion of male individuals increased with increasing COVID-19 severity (Fig 1B and 1C and S2 Table).

The European subcohort, DeCOI_EUR_, comprised 1,017 individuals (WHO 1–3: n = 362; WHO 4–5: n = 383; WHO 6–10: n = 272, S2 Fig). Again, the average age and proportion of male individuals increased with COVID-19 severity (Fig 1B and 1C and S2 Table). For association analyses in DeCOI_EUR_, we created two case-control definitions: (i) “extreme” (Ex / cases: hospitalized severe, n = 272 / controls: ambulatory mild, n = 362), and (ii) “all_hospitalized” (B1 / cases: hospitalized moderate and hospitalized severe, n = 655 / controls: ambulatory mild, n = 362), with B1 being in accordance with the case control definition of the COVID-19 HGI and Ex representing the analysis along the phenotypic extremes.

### Targeted analysis of variants at the *TLR7* locus

Given that some monogenic disorders are likely to impact the course of COVID-19 disease [34], the multi-ethnic DeCOI cohort was analyzed for the presence of known monogenic diseases. We first queried for variants that may cause *TLR7* deficiency, since at the time of writing, this represents the most robustly established monogenic cause of severe COVID-19, particularly in young men [14,15,19]. Within the coding sequence of *TLR7*, three known variants were identified (S5 Table). Each of these variants had low REVEL/CADD scores. Carriers were observed in all phenotypic categories, which is consistent with the normal functional characteristics of these three variants, as described elsewhere [15]. Within non-coding regions with evidence for regulatory function (see Methods), 23 variants with an MAF < 1% were identified across all phenotypic groups (S5 Table). The most notable variant was rs192357402, which was observed in 3/199 severely affected males of European-ancestry but was not detected in 391 males of European-ancestry with non-severe disease (p = 0.038, Fisher’s exact test). This finding was not replicated in 672 males of European ancestry in an independent dataset from the Biobank Quebec COVID-19 Cohort (Methods, 1/113 severe vs. 2/559 non-severe; p = 0.42, S6 Table). Based on coverage data in the DeCOI cohort VCF, a search was also conducted in males for evidence of deletions within a region spanning approximately 200kb centered around *TLR7*. While 57 individuals were found to have short stretches of missing coverage, visual inspection provided no evidence that these were true deletions.

### Analysis of 13 genes previously implicated in severe COVID-19

Previously, deleterious variants in 13 genes of the type I interferon (IFN) immunity were implicated in life-threatening COVID-19 pneumonia [11]. We queried these genes for variants predicted to be loss-of-function (pLoF), as well as for missense variants previously demonstrated to be LoF or strongly hypomorphic (see Methods). Six heterozygous pLoF variants in the genes *UNC93B1*, *IRF7*, *IRF3*, *IFNAR1* and *IFNAR2* and two heterozygous missense variants in *IRF3* and *IRF7* (S7 Table) were identified. Interestingly, one moderately affected male aged 25–34 years carried two of these variants (IFNAR2/pLoF and IRF3/missense). The carriers of these variants were 46.1±15.8 years old on average (p = 0.13, Student’s t-test, comparison against the remainder of the DeCOI cohort), three of the seven individuals were female. Only one individual was severely affected, three were moderately and three were mildly affected, which indicates that the phenotype of these individuals is not more severe than expected by chance (expected number of individuals by random chance: 2.0 severe, 2.8 moderate and 2.2 mild). No homozygous or potentially compound heterozygous variants that passed our filter criteria were identified. Systematic testing for joint association of variants within the 13 genes of the type I IFN immunity can be found below.

### Targeted analysis of monogenic disorders

Next, the DeCOI cohort was queried for the presence of established causes of monogenic diseases, as based on variants reported in ClinVar. Autosomal-recessive (AR), autosomal-dominant (AD) and X-linked (XL) patterns of inheritance were considered (see Methods). Established homozygous variants causing monogenic disorders were found in 4 out of 1,220 individuals, and likely compound-heterozygous variants were identified in two individuals (jointly 0.5%, Table 1). All six individuals were male and hospitalized (3/6 with a fatal course). Notably, the six individuals were significantly younger on average than the remainder of the DeCOI cohort (mean±SD = 38±14.5yrs; p = 0.027, Student’s t-test; S3 Fig). Heterozygous variants with established associations to dominantly inherited monogenic diseases, and that are annotated as “pathogenic” or “likely pathogenic” in ClinVar, were present in 77 out of 1,220 DeCOI individuals (6.4%). The associated diseases covered a broad range of categories, with endocrine, hematologic, and ophthalmologic disorders being the most commonly represented (Fig 1D). Overall, carriers of heterozygous (likely) pathogenic ClinVar variants did not differ significantly from the rest of the DeCOI cohort with respect to sex, age, or severity of COVID-19 (S3 Fig). No hemizygous or homozygous variants on the X-chromosome were identified that are annotated as “pathogenic” or “likely pathogenic” in ClinVar.

**Table 1:** Characteristics of carriers of homozygous or likely compound heterozygous disease variants in the DeCOI cohort.

Gene	Variant / Genotype	Monogenic disease	Sex, age range	COVID-19 severity	Monogenic disease previously reported	Additional information	Population background
*BBS1*	Homozygous splice variant: chr11_66523577_G_A; c.951+1G>A;p.?	Bardet-Biedl syndrome 1	Male, 35–44 years	Fatal (WHO 10)	no	Clinically intellectual development disorder, blindness, and seizures	AMR
*AGXT*	Homozygous frameshift variant: chr2_240868890_A_AC; p.Lys12GlnfsTer156	Primary Hyperoxaluria Type 1	Male, 25–34 years	Fatal (WHO 10)	yes	Post renal and liver transplant status (no details available concerning immunosuppressive therapy)	EUR
*SERPIN1C*	Homozygous missense variant: chr1_173914743_G_A; p.Pro73Leu	Antithrombin Budapest 3	Male, 35–44 years	Moderate (WHO 4)	not available	-	SAS
*AIRE*	Homozygous nonsense variant: chr21_44289773_C_T; p.Arg257Ter	Polyglandular autoimmune syndrome	Male, 15–24 years	Severe (WHO 6)	yes	-	AMR
*HBB*	Likely compound heterozygous variants: Intron variant chr11_5225832_G_C; NM_000518.5:c.316-106C>G Nonsense variant chr11_5226774_G_A; p.Gln40Ter	Beta- thalassemia major	Male, 35–44 years	Moderate (WHO 4)	not available	-	EUR
*PAH*	Likely compound heterozygous variants: Missense variant chr12_102843676_T_C; p.Glu390Gly Missense variant chr12_102855313_C_G; p.Val177Leu	Mild Phenylketonuria (PKU)	Male, 55–64 years	Fatal (WHO 10)	no	Heart disease and diabetes mellitus type 2	AMR

Abbreviations: AMR: Admixed American; EUR: European; SAS: South Asian. Note that the genomic position is given in GRCh38 coordinates.

### Gene- and gene-set-based collapsing analyses

Next, the analyses were expanded to study joint effects of rare variants across: (i) single genes, and (ii) sets of genes with presumed importance to COVID-19 (see Methods, S3 and S4 Tables). For this purpose, variants were selected on the basis of allele frequency and predicted functional effect, and all variants were collapsed across a gene or a gene-set. Association testing was then performed with logistic regression, including polygenic score based on common variants as one covariate in addition to principal components (PC) and age-/sex-derived measures (see Methods for more details). Results of the gene-based collapsing analyses are shown in S8 Table for analysis Ex, and S9 Table for analysis B1. Some nominally significant results were observed. However, these did not withstand correction for multiple testing, and their number was not larger than would be expected by chance (S4 Fig).

**Fig 2:**
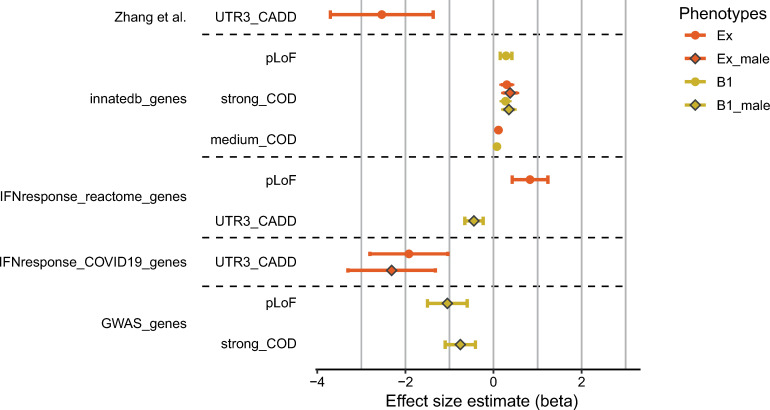
Effect sizes of nominally significant gene-set based tests in the DeCOI_EUR_ cohort. Gene-sets and the corresponding functional masks (S4 Table) that were tested are given on the y-axis. On the x-axis, effect size estimates (betas) are shown as markers with error bars indicating the standard errors of betas. Note that phenotypes are color-coded, and the markers outlined in black indicate analyses that only included males. Nominally significant findings were only obtained in the overall analyses and male sub-stratification. None was observed in female-only or age-stratified analyses. A list of genes that were included in each gene-set can be found in S3 Table.

The gene-set analyses were performed on the case-control definitions Ex and B1 overall, and then as stratified according to sex (male/female) and age (younger than 60 years/older or equal 60 years). In total, 14 nominally significant phenotype / gene-set / mask combinations were identified, all of which were observed in either the overall phenotypes (Ex_all/B1_all) or the male subcohort (Ex_male; B1_male; Fig 2 and S10 Table). None of the other stratifications (female or age-stratified) yielded any significant enrichment. Nominally, the most significant enrichment was found among severe COVID-19 patients in genes of the innate immune system, for the functional masks (FM) that included predicted loss-of-function (pLoF) (B1_all: p = 5.85x10^-03^; beta = 0.27, SE = 0.099) and pLoF+missense (Ex_all: p = 7.04x10^-03^; beta = 0.11, SE = 0.042). Among the non-coding variants, a nominally significant depletion of 3’UTR variants with high CADD scores (CADD≥10) was observed in both gene sets related to IFN-response (Ex_male/IFN_response_COVID-19/UTR3_CADD: p = 0.019; n = 31 genes), and the subset of 13 genes with *a priori* evidence for an involvement in severe COVID-19 (Ex_all/Zhang et al./UTR3_CADD: p = 0.029). In the gene-based analyses that did not include individual PRS as a covariate, highly correlated results were generated (S4 Fig).

### Single variant association analyses

**Fig 3:**
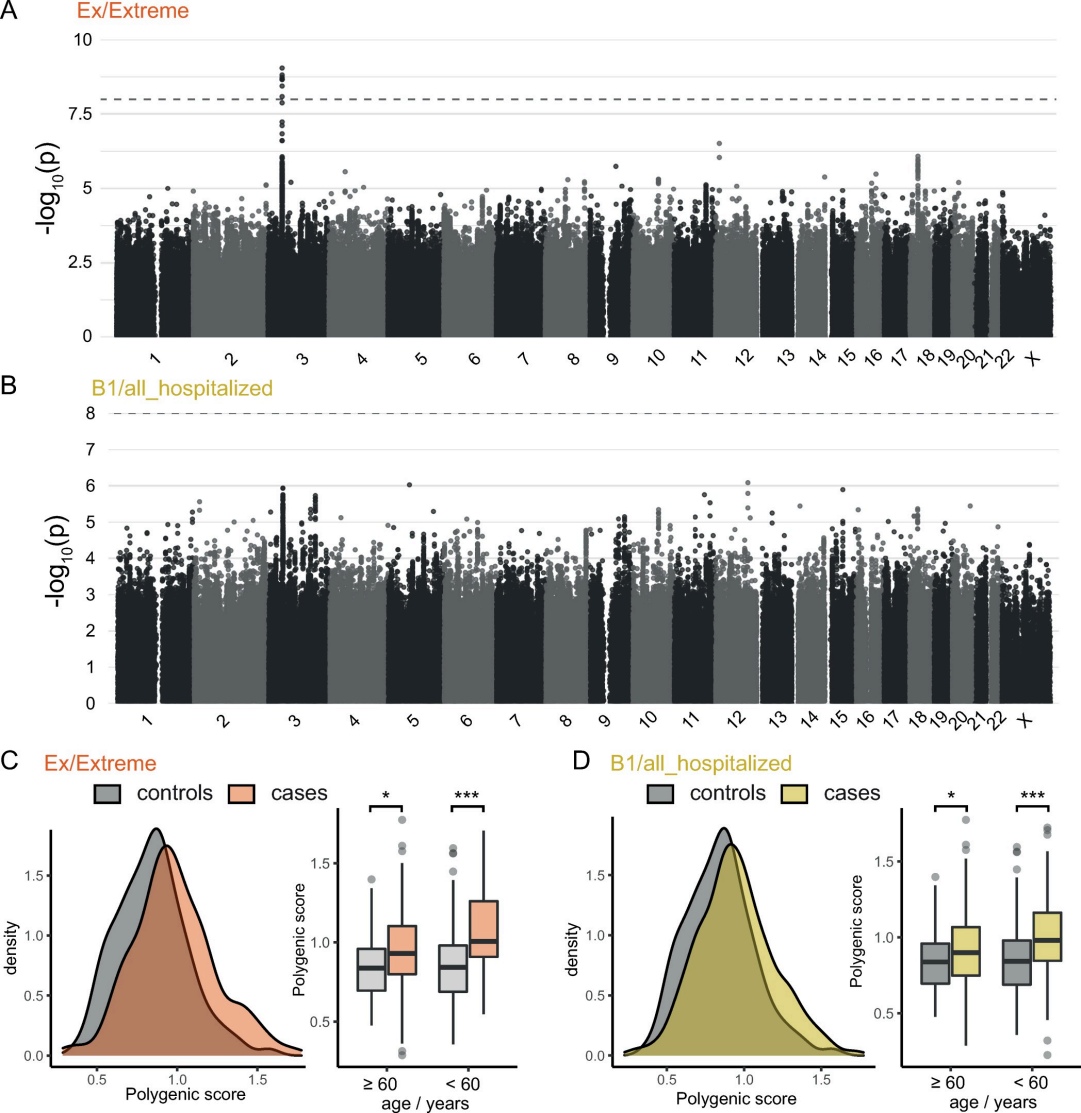
Analysis of common variants within the DeCOI_EUR_ cohort. (A) and (B): Manhattan plots of association analyses of single variants (MAF>0.5%) in DeCOI_EUR_ (n = 1,017 individuals), for phenotype Ex (272 severely affected individuals vs. 362 mild controls) and B1 (655 hospitalized individuals vs. 362 non-hospitalized controls), respectively. Genomic inflation factors were 1.04 (Ex) and 1.00 (B1). Among the strongest associations is the well-established risk locus at 3p21.31. Panels (C) and (D) show the distribution of individual polygenic risk scores (PRS) among cases (orange or yellow) and controls (gray) of Ex (C) or B1 (D) overall (density plots in the left parts) or when stratified according to age below or above 60 years (box plots in the right parts). The elements of the box plots correspond to the following values: thick line: median, box: 25th and 75th percentile, whiskers: largest / smallest value not further away from the box than 1.5 times the interquartile range, points: values outside of the range of the whiskers. *: p<0.05, ***: p<0.001; Wald test followed by Bonferroni correction. MAF: Minor Allele Frequency.

After analyzing lower frequency variants, we next investigated more common variants. Using WGS genotype calls, GWAS were performed for phenotypes Ex and B1, respectively (Figs 3 and S5). Interestingly, despite the relatively low sample size of the Ex case-control definition, association reached genome-wide significance for variants at the established key risk locus 3p21.31. In analysis Ex, 177 variants with p<1x10^-05^ were observed at 19 loci, the majority of which (n = 128) mapped to the 3p21.31 region (S11 Table). The variant with the strongest evidence of association was rs73064425 (chr3:45859597:C:T, p = 9.00x10^-10^; beta = 1.44, SE = 0.23). In Europeans, this variant is in perfect LD with all previously reported lead variants (i.e., rs11385942 [9], rs10490770 [35], and rs35044562 [36]). No additional support for any of the 49 variants outside 3p21.31 was found in data from the WGS-based summary statistics from GenOMICC [22] or the array-based GWAS of the COVID-19 HGI (release 7, without GenOMICC [10], S11 Table). At established risk loci for SARS-CoV-2 related traits (n = 71) [7–10], nominal significance was observed for the reported lead variants at 11 loci (Tables 2 and S12), whereby a minor overlap of samples between COVID-HGI and DeCOI (<0.04%) must be kept in mind. No significant association was found for two variants that were reported to be associated with severe COVID-19 in previous independent German cohorts (i.e., rs5443 (p = 0.72 (Ex) and p = 0.14 (B1)); and rs5010528 (p = 0.41 (Ex) and p = 0.77 (B1))) [37,38]. Finally, the DeCOI_EUR_ cohort was stratified according to age or sex, and the better-powered Ex analysis was repeated for different substrata. No variants in any of the stratified analyses reached genome-wide significance (S6 Fig and S13 Table).

**Table 2:** Previously reported risk loci for COVID-19 with nominal significance in DeCOI_EUR_.

Chr	Pos	ID	Ref/Alt	Extreme (272 cases^a^, 362 controls)^1^			All_Hospitalized COVID-19 (655 cases^b^, 362 controls)			Candidate gene(s)	Ref (PMID)
				beta	SE	P-value	beta	SE	P-value		
1	9067157	rs2478868	A/C	**0.34**	**0.15**	**0.025**	**0.36**	**0.12**	**0.0021**	SLC2A5	37198478
1	77501822	rs71658797	T/A	0.51	0.26	0.050	**0.59**	**0.20**	**0.0041**	AK5	37198478
1	155197995	rs41264915	A/G	**-0.78**	**0.25**	**0.0015**	**-0.75**	**0.19**	**0.00011**	THBS3, MUC1	35922517
3	45818159	rs17713054	G/A	**1.39**	**0.23**	**0.0000000021**	**0.84**	**0.19**	**0.0000091**	LZTFL1, CXCR6	32558485
4	25312372	rs16877005	A/G	**0.74**	**0.37**	**0.048**	0.49	0.27	0.075	PI4K2B	37674002
4	167824478	rs1073165	A/G	**0.29**	**0.14**	**0.0361**	0.075	0.11	0.51	DDX60	37198478
10	112972548	rs7897438	C/A	-0.33	0.18	0.061	**-0.28**	**0.14**	**0.044**	TCF7L2	37674002
11	34482745	rs61882275	G/A	**-0.37**	**0.15**	**0.012**	**-0.39**	**0.12**	**0.00079**	ELF5	35255492
19	10305768	rs73510898	G/A	**0.71**	**0.27**	**0.010**	**0.55**	**0.21**	**0.0092**	ZGLP1, RAVER1, ICAM5	3525549233307546
19	10414696	rs142770866	G/A	0.53	0.27	0.051	**0.47**	**0.21**	**0.028**	PDE4A	37198478
19	48867352	rs4801778	G/T	**-0.41**	**0.19**	**0.032**	**-0.35**	**0.15**	**0.020**	PLEKHA4, TULP2	34237774

Bold if nominally significant in the respective analysis.

^a^WHO-scores 6–10.

^b^WHO-scores 4–10, corresponding to the B1 phenotype definition of COVID-19 HGI. Abbreviations: Chr: Chromosome; Pos: Position in GRCh38 coordinates; ID: rs-ID of the SNP; Ref: Reference allele; Alt: Alternative allele;SE: Standard error; Ref (PMID): Reference given as PubMed ID.

### Autozygosity

To investigate a possible effect of autozygosity on disease severity, inbreeding coefficients were calculated as a measure for autozygosity within the DeCOI_EUR_ cohort, with no prior filtering of variant frequency. For phenotype Ex, no significant differences in autozygosity levels were observed. Significantly increased inbreeding coefficients were observed in cases of phenotype B1 (cases: mean±sd: 0.002±0.01; controls: 0.001±0.005; p = 0.023, one-sided Wilcoxon test; S7 Fig). This result was mainly driven by a small subset of individuals with inbreeding coefficients above 0.02 (FI>0.02: 3.51% in cases, 0.83% in controls; FI>0.05: 0.76% vs. 0.15%; FI>0.1: 0.55% vs. 0.0%), who largely overlapped with samples that were located outside of the central European-ancestry cluster on the PC plot (S8 Fig). When the first 10 PCs were added as covariates to a logistic regression in order to capture population substructure, the above results became non-significant (p = 0.55, Wald test). Prior filtering of variants with MAF <1% rendered the difference between cases and controls non-significant (p = 0.068, one-sided Wilcoxon test).

### Polygenic risk scoring

Next, analyses were performed to investigate whether the aggregated effect of common variants in PRS was significantly increased in cases compared to controls in Ex and B1, and whether the effect differed across age groups. Using PRS generated for individuals within the DeCOI_EUR_ cohort on the basis of the GenOMICC study [22], a significantly larger PRS was observed in cases compared to controls for both phenotypes (p<0.001, Wald test followed by Bonferroni correction of p-values). Upon age stratification (younger than 60 years/older or equal 60 years), this result became even more pronounced, with higher mean PRS values being observed in younger cases than in older cases (Fig 3 and S14 Table; <60 years: p(Ex)<0.001, p(B1)<0.001; ≥60 years: p(Ex) = 0.009, p(B1) = 0.035, Wald test followed by Bonferroni).

Analyses were then performed to determine whether the inclusion of PRS improved the approximation of the present data by logistic regression models. For this purpose, two logistic regression models were fitted: 1) with covariates only (namely sex, age, age^2^, age*sex and the first 10 PCs derived from common variants); and 2) with the same covariates and PRS. When PRS were added, a significant increase in Nagelkerke’s R^2^ was observed (Ex: from 0.466 to 0.504; p = 1.34x10^-7^; B1: from 0.403 to 0.424, p = 1.85x10^-6,^ likelihood-ratio test). Analyses were then performed to test whether the addition of PRS to the covariates improved the prediction of hospitalization or a severe disease course. The dataset was split at random 1,000 times into test and training sets, and logistic regression models were fitted to the training set (see Methods). Areas Under the Curves of the Receiver Operating Characteristic curves (AUROCs) were then determined on the test sets. In 1,000 splits, AUROCs were higher (on average) for the model that included PRS, and the median increase of AUROCs was 0.022 (minimum: -0.200, maximum: 0.263) for the hospitalization (B1) and 0.056 (minimum: 0.033, maximum: 0.078) for the extreme (Ex) case-control definition.

## Discussion

The present report introduces the DeCOI cohort as one of only a few WGS datasets of 1,000 or more SARS-CoV-2 positive individuals worldwide. While we did not detect any causal variant in or around the established risk gene *TLR7*, the analyses identified carrier status for six autosomal-recessive monogenic disorders in young males who had been hospitalized due to COVID-19. In the European subset (DeCOI_EUR_), burden testing revealed nominal enrichments of rare variants in coding and non-coding regions of genes that are implicated in the interferon immune response both in the cohort overall and in the male-only subgroup. The present analyses also confirmed associations between previously reported common risk loci and COVID-19 severity, including a genome-wide significant association for the risk locus at 3p21.31, and showed that their aggregation into PRS accurately captured risk in an age-dependent manner. Besides complementing ongoing, systematic COVID-19 host genetic efforts to study common [7–10] or rare variants [11,12,14,17,18], our study can be used to jointly analyze variation across the entire frequency spectrum as part of larger, multi-study efforts.

The largest WGS study on severe COVID-19 to date was performed by GenOMICC, and focused on critically-ill patients from intensive care units [22]. This study included more than 7,400 individuals with severe COVID-19, and rare variant associations were analyzed using standard gene-based approaches [22]. Here, the DeCOI WGS data were explored in additional dimensions, including analyses performed from a clinical genetics perspective. Although our sample size was limited, two characteristics of the DeCOI cohort rendered it suitable for the present analyses. First, the cohort included SARS-CoV-2 infected individuals with mild disease who could be used as controls. The presence of rare causal risk variants among these controls was unlikely, thereby increasing confidence in the rare variant results. Second, the vast majority of participants were recruited during the first 12 months of the pandemic, when: (i) most individuals were not vaccinated against SARS-CoV-2; (ii) re-infections were uncommon; and (iii) SARS-CoV-2 diversity was still low. On the other hand, use of the WHO classification system as a proxy phenotype for severity likely increased classification heterogeneity ‐ this might have limited our statistical power. We envision that the robust identification of low-frequency and rare risk variants will require large cohorts, which is supported by the fact that the GenOMICC consortium failed to identify rare individual genetic factors at the level of genome-wide significance, despite their relatively large sample size and homogenous phenotype definition. Further, additional factors such as prior stimulation of the immune system through viral infections [39] and/or vaccination [40], or the presence of type-I-interferon autoantibodies [3,41], also shape the immune response of each individual, and contribute to the clinical outcomes of SARS-CoV-2 infections. Therefore, future approaches involving the integration of genetic data with clinical information on immune related traits and multi-omics data could facilitate elucidation of the etiological landscape of COVID-19. Notably, such information (e.g., single-cell transcriptomics [42,43]) is already available to some extent for the DeCOI cohort and will be used for subsequent integrative analyses.

Studies that identified TLR7 deficiency as a monogenic form of severe COVID-19 [14,15,19] were limited to the *TLR7* coding region, and thus did not consider potential causal variants in adjacent regions with evidence of regulatory function (including structural variants). Despite comprehensive analyses, no causal SNVs or small indels were detected in the DeCOI cohort, neither in coding nor non-coding regions. This included a lack of any potential causal deletion at the *TLR7* locus in males, which we investigated using coverage data. Nevertheless, the analysis suggested the overrepresentation of a low-frequency variant, located in a constitutive enhancer element that was identified by ENCODE, in severely affected men. However, this result could not be replicated in a small independent WGS dataset, and thus remains inconclusive. We also investigated the association between variants in additional 13 genes of type I interferon (IFN) immunity, for which a recent study estimated a joint odds ratio of 3.11 [95% confidence interval (CI) 1.4–8.6] for having life-threatening COVID-19 when carrying heterozygous pLoF variants in these 13 genes [20] (reported allele frequency of pLoF variants within the 13 genes: 0.004). In our cohort, we identified 7 carriers of at least one heterozygous variant in 5 of these genes but the mutation carriers did not show more severe disease courses than expected by random chance, in line with the absence of replication in other clinically heterogeneous cohorts [17,18,22,44]. Interestingly, in our study we observed an odds ratio of 4.03 for the common lead variant at 3p21.31 (Ex, rs17713054, 95% CI: 2.56–6.37, MAF: 0.08). We speculate that in our cohort, the relevance of monoallelic (i.e. heterozygous) deleterious variants in the 13 genes of the type I IFN immunity is limited. However, this does not exclude the possibility that biallelic variants resulting in rare autosomal recessive inborn errors of immunity within these genes could underlie unexpectedly severe cases, such as severe COVID-19 in children, in the German population, for which our dataset was underpowered.

Epidemiological evidence suggests that pre-existing conditions are a major risk factor for severe COVID-19 [2,34]. The present analyses identified six recessive monogenic disorders in male individuals, who had presented with severe or moderate COVID-19. While this does not imply any causality, it is of note that these six individuals had an age that was below the average age of the DeCOI cohort overall. In several of these individuals, a modification of the COVID-19 phenotype by the underlying monogenic disease is biologically plausible (see S1 Text). For example, biallelic variants within *AIRE* can cause autoimmune polyendocrinopathy syndrome type 1 (APS-1). In individuals with APS-1, antibodies against IFN-ɑ and IFN-⍵ are frequently present, and moderate or severe COVID-19 has been described in SARS-CoV-2 infected APS-1 patients [45–47]. Additionally, some of the recessive diseases identified lead to an impairment of important organ systems and could therefore indirectly predispose to more severe COVID-19 disease outcomes (see S1 Text), e.g. Bardet-Biedl Syndrome 1 probably caused the intellectual developmental disorder, and primary hyperoxaluria might have been responsible for the kidney and liver transplant in the two study participants who died of COVID-19, respectively.

In contrast to individuals with putative autosomal-recessive disorders, individuals with putative autosomal-dominant disorders did not differ from the remainder of the DeCOI cohort regarding age or COVID-19 severity. This could be due to a lack of power, which might be attributable to factors such as reduced penetrance, which is more common in dominantly inherited disorders [48]. Overall, it needs to be kept in mind that the results for both autosomal recessive and autosomal dominant monogenic disorders are from a non-representative sample and insufficient to establish any causality.

At the single-gene level, no significant enrichment of rare variants was observed beyond that which would have been expected based on chance alone. Furthermore, the gene-set based analysis of rare variants across candidate genes only yielded nominally significant results. The lowest p-values in our gene-set based analysis were generated for genes that are implicated in the innate immune system, specifically the IFN pathways. Here, pLoF variants, either alone or in combination with missense variants, were enriched in hospitalized or severely affected individuals. Surprisingly, we also observed nominally significant enrichments in mild COVID-19, of variants in the 3’UTRs of genes from the interferon pathway and at GWAS loci. While these results do not withstand statistical correction and warrant independent replication, they are complementing a recent study which identified a highly significant depletion of 3’UTR variants in the gene *IL18RAP* in amyotrophic lateral sclerosis (ALS) patients [49]. Specifically, for IFN genes, we speculate that 3’UTR variants might contribute to an increased stability or abundance of gene product, e.g. through abolishment of miRNA binding sites, as recently suggested for a 3’UTR variant in *TRIM14*, a gene also implicated in the type I IFN pathway [50]. In the gene-/gene-set based collapsing analyses, the availability of the individual’s common genotypes was leveraged in order to weigh down individuals with higher PRS, as it has been suggested that integration of PRS into rare-variant burden analyses might be beneficial in terms of their statistical power [51]. It is important to note that most of the rare variant burden signals in the present study were driven by male individuals, which suggests the presence of sex-differences in terms of the extent to which rare variants contribute to severe COVID-19 risk. This finding requires replication in independent cohorts. Also, in the future, novel statistical models that include variants spanning the entire frequency spectrum may enhance the power for rare variant and/or gene identification in cohorts such as DeCOI. A subsample of the present DeCOI cohort already contributed to one such effort [28].

Interestingly, despite our relatively small cohort size, in the association analysis of more frequent variants, our analysis found a comparably large effect size for the contribution of the known risk locus at 3p21.31 to COVID-19 severity, resulting in genome-wide significance. This indicates that this locus is relevant to our cohort of mainly German individuals which might also be true to the German population. Additionally, previously reported GWAS signals were replicated at nominal level, despite a sample size that was substantially lower than those of the discovery cohorts (i.e., GenOMICC or COVID-HGI) [10,22]. When common variants were aggregated into PRS and applied to overall and age-stratified groups, a larger genetic contribution of common genetic variation to COVID-19 severity was observed in younger individuals. While this has been described previously for candidate lead variants at individual major risk loci [35,52], the present study expanded this analysis to the genome-wide scale. In older individuals, the addition of PRS for COVID-19 severity only moderately improved predictive models, as shown in data from the UK Biobank alone [53] or in the UK Biobank plus three additional US-American cohorts [27]. Since neither of these two studies performed age-stratified analyses, our data suggest that the addition of genetic factors to predictive models could prove particularly helpful in younger individuals, and highlight the translatory potential of PRS. Importantly we constructed the PRS on the basis of WGS data from the GenOMICC cohort, thus reducing the impact of technical variation on score construction.

In conclusion, while the performance of WGS studies continue to be hampered by considerations of cost and sample size, this flagship analysis of the DeCOI cohort highlights the potential of WGS in terms of both investigating variants that are inaccessible to other methods, and performing combined analyses of variants from the entire allelic spectrum, respectively. A more complete understanding of the underlying genetic architecture will be of paramount importance to the clinical (risk) management of individuals with COVID-19 and its post-acute sequelae, which are likely to play important roles in quotidian clinical practice for years to come.

## Methods

### Ethics statement

Written informed consent for host genetics analyses was obtained from each participant or their legal representative in case of minors. The study received ethical approval by the Ethical Review Board (ERB) of each participating center: Faculty of Medicine at Technical University Munich (TUM 217/20, TUM 221/20S, TUM 440/20S); Medical Faculty of the University Bonn (Approval Nr. 171/20 and 468/20); University of Cologne (20–1295); University Hospital Cologne (160054 and 2001187); Landesärztekammer des Saarlandes (62/20); Medical Faculty of the University Hospital Tübingen (Approval Nr. 286/2020B01); University Hospital RWTH Aachen (EK 080–20); University Hospital Essen (UME: 21-9900-BO); Medical Faculty of Goethe University Frankfurt am Main (20–748); Healthcare System of the Autonomous Province of Bolzano; Medical Faculty of Heinrich-Heine-University Düsseldorf (5350 ‐ amendment for COVID19); Hannover Medical School (9001_BO_K); LMU University Hospital Munich (20–245); Medical Faculty of the LMU Munich (20–263); and Medical Faculty of the University of Regensburg (20-1785-101). Additional details on ERBs are provided in S1 Table.

### Recruitment of participants

DeCOI was founded in the spring of 2020, with the aim of advancing next-generation sequencing (NGS)-based COVID-19 research in the areas of viral epidemiology, functional genomics, and host genetics [32]. For the host genetic analyses participants were recruited at 16 different sites, 15 of which were situated in Germany, and one in the German-speaking region of Italy (South Tyrol), from individual COVID-19 studies that were being conducted at the respective institutions. The inclusion criteria for the host genetics analyses were: (i) available DNA; (ii) a test-confirmed SARS-CoV-2 infection; and (iii) explicit consent for WGS analysis. Notably, the type of test used for confirmation of a SARS-CoV-2 infection (self-reports based on rapid antigen tests and/or qPCR) varied across the 16 recruitment sites. Descriptions of the individual studies are provided in S1 Table.

We included 1,275 individuals for WGS analysis. The minimum phenotypic dataset for each individual that was available to the research team comprised sex, age, and information on COVID-19 disease course in accordance with the World Health Organization (WHO) ordinal scale [33]. The majority of individuals (n = 1,204; 94.4%) were infected in 2020 (n = 1,136/1,275; 89.1%) or early 2021 (January to April 2021, n = 68; 5.3%) and therefore were naive for any COVID-19 vaccination at the time of reported infection. For 71 individuals, no information on vaccination status was available. However, given the limited population-wide availability of COVID-19 vaccination during 2021, and the fact that the latest time point of reported infection in these cases was December of 2021, these individuals are unlikely to have been vaccinated at the time of recruitment.

### WGS data generation

Library preparation and sequencing was performed using consolidated workflows at three different sites of the German NGS Competence Centers, i.e., the Cologne and Bonn sites of the West Germany Genome Center (WGGC), and the NGS Competence Center Tübingen (NCCT). In brief, genomic DNA was quantified using the Qubit dsDNA HS assay kit and a Qubit fluorometer (ThermoFisher). DNA library preparation was performed using the TruSeq DNA PCR-Free kit (Illumina), in accordance with the manufacturer’s instructions. Up to 1.2 μg of genomic DNA was fragmented to 350 bp using ultrasonication on the LE220 focused-ultrasonicator (Covaris). The resulting libraries were sequenced as paired-end 150 bp reads on an Illumina NovaSeq6000, with a sequencing output of approximately 120 Gb per sample.

At each sequencing site, demultiplexing and FastQ file generation was performed using bcl2fastq2 version 2.20.0.422, and quality control (QC) statistics were generated using FastQC v0.11.9. Subsequently, sequencing reads were aligned to the human reference genome (GRCh38), duplicates were removed, and single nucleotide variants (SNVs) as well as short indels were called using the Illumina DRAGEN platform (software version 3.5.7 or 3.6.3). The resulting gVCF files were transferred to the study analysis hub (WGGC_Bonn), and joint variant calling of all samples was performed using a slightly modified version of GLnexus v1.3.1 (setting: “gatk”) in order to yield a raw cohort VCF (“raw-cVCF”). Modifications to the standard GLnexus pipeline included community changes that optimize the caller for haploid regions, which are reported differently in GATK and DRAGEN.

### WGS data analysis

The raw-cVCF was modified in order to retain biallelic variants with high-quality individual genotypes only. For this purpose, individual genotypes were set to “missing” if they had low coverage (sequencing depth (DP) < 4 reads) or a genotype quality (GQ) < 20. Furthermore, genotypes were only retained if the fraction of reads with alternative alleles was <10% or >90% for homozygous or hemizygous positions, or between 25% and 75% for heterozygous positions. Based on this list of high-quality variants (“cVCF”), two variant sets were established by applying additional filters. The first variant set was termed *“Common variants for QC”* (n = 452,867). Here, the variant set was restricted to variant calls with a minimum DP of 8, a minimum variant call rate (vCR) of 95%, and a minor allele frequency (MAF) >1%. Variants were then limited to those outside of regions with high linkage disequilibrium^32^ (LD; see URL section), and were pruned (r^2^: 0.2, window size: 1Mb). The second variant set was termed *“Generic variant set”* (n = 53,195,313). Here, after removing samples that did not pass sample QC (see below), calls with DP<8 were set to missing in all genomic regions of females and in autosomal/pseudoautosomal (PAR) genomic regions of males. In addition, heterozygous calls in non-PAR regions of males were set to missing, and only variants with a vCR above 95% were retained.

Functional annotation of variants *in silico* was performed using: (i) the command line version of Variant Effect Predictor (VEP; version 101) with the plugin TSSDistance; (ii) the external annotation sources gnomAD (version 2.1.1 as well as 3.1.2), ClinVar (version 20221008), dbNSFP (version 4.1a), CADD (version 1.6), SpliceAI and core regions of DNAse I hypersensitive sites (see URLs). The option “pick_allele_gene” was used to ensure that only one consequence per gene was reported for each variant allele.

### Sample QC and population subcohorts

Of the 1,275 samples, 35 had an average coverage of <20x and/or a call rate of <90% (based on the “*common variants for QC*” set and autosomal regions, S1 Fig), and were therefore excluded. Next, a subset of the “*common variants for QC*” (Hardy-Weinberg p-values above 0.001 in presumed females) was used to determine genetic sex via the check-sex function of PLINK (version 1.9). Here, 20 individuals were excluded due to divergent genotypic and phenotypic sex. This resulted in a final set of 1,220 individuals (“DeCOI cohort”; Fig 1A and 1B and S2 Table) with diverse population backgrounds.

For the formal statistical analyses, a homogeneous subset of unrelated individuals from one major population background was generated using the “*common variants for QC*” variant set and data from the 1000 genomes project [54]. Principal component (PC) analysis was conducted on variants that were common to both datasets using PLINK (version 1.9). Based on the obtained PCs and the population annotations within the 1000 genomes project, individuals in the DeCOI cohort were then assigned to continental populations. To determine relatedness, kinship coefficients were calculated using the KING software (version 2.2.7). Individuals were defined as related when they had kinship coefficients > 0.04, which indicates third-degree relatedness or closer. From each pair of related individuals, the least severely affected individual was excluded. This approach resulted in a cohort of 1,017 unrelated individuals from the European population (“DeCOI_EUR_”; Fig 1A and 1B and S2 Table). Due to the low number of individuals of non-European ancestry, no other population subcohort was suitable for association testing.

### Case/control definitions for association analyses

On the basis of the available phenotypic information, the study participants were classified as having one of three phenotypes: “ambulatory mild” (WHO 1–3), “hospitalized moderate” (WHO 4–5), or “hospitalized severe” (WHO 6–10). For association analyses, these classes were used to assign case/control status to 1,017 individuaIs of the DeCOI_EUR_ cohort, for two separate case/control definitions (Fig 1A and 1B): (i) “extreme” (Ex / cases: hospitalized severe, n = 272 / controls: ambulatory mild, n = 362), and (ii) “all_hospitalized” (B1 / cases: hospitalized moderate and hospitalized severe, n = 655 / controls: ambulatory mild, n = 362). The phenotype B1 is in accordance with the definition by the COVID-19 HGI [8].

### Targeted analysis of variants at the TLR7 locus

The following SNVs were retrieved from the raw-cVCF: (i) those located within *TLR7* protein-coding regions; and (ii) those located in the promoter, 3’/5’ untranslated regions (UTRs) and regions annotated as SCREEN enhancers by the ENCODE project (accessed November 30, 2022; 13 elements within the gene body and 50 kb upstream of the transcription start site (TSS)). For the protein-coding regions, the following were selected: (i) all putative loss of function (pLoF) and non-synonymous variants (VEP impact “high” or “moderate”); and (ii) variants with potential effects on splicing (defined as “any spliceAI delta score above 0.5”), independent of MAF. For the non-coding regions, variants were included if they had a maximum allele frequency of 1% according to gnomAD v3.1.2 (popmax value). To identify potential deletions at the *TLR7* locus, the cohort VCF (region: chrX:12760551–12980636) was queried for stretches of 3 or more variant positions with missing coverage in male individuals.

### Filtering for rare variants with strong effects according to variant effect predictions or ClinVar

To identify rare variants with strong effects in DeCOI, we selected variants with an allele count of <5 within the cVCF (n = 1,220 individuals), and excluded variants that had more than one homozygous report in any population from either gnomAD exomes (version 2.1.1) or gnomAD genomes (version 3.1.2). For variants in genes linked to dominant Mendelian disorders, an allele count of 50 or below in gnomAD exomes or genomes was required (sum across all population backgrounds, respectively).

For homozygous or hemizygous variants, a ratio between alternative and total reads (allelic balance) of higher than 95% was required. For heterozygous variants an allelic balance between 25% and 75% was required, as well as a read count of at least 4 for both the reference and the alternative allele. To identify potential compound heterozygous variant carriers, we first filtered for individuals with ≥ 2 variants in the same gene. Subsequently, variant co-occurrence (gnomAD version 2; [55]) and/or review of the literature was used to determine if the variants are likely affecting one allele (in *cis*) or both alleles (in *trans*, i.e. compound heterozygous). Based on this strategy, the following analyses were performed:

- For the “*Analysis of 13 genes previously implicated in severe COVID-19*” we only considered variants that were predicted to be LoF (VEP impact “high”) or that were previously shown to result in functional alterations [11].
- For the “*Targeted analysis of monogenic disorders”*, we only retained variants reported as being pathogenic or likely pathogenic in ClinVar by multiple submitters or by expert panels (version 20221008, n = 40,189) [56]. Variants within genes from the American College of Medical Genetics and Genomics (ACMG) secondary findings list [57] were excluded, and variants were only retained if they affected a gene annotated with an Online Mendelian Inheritance in Man (OMIM) phenotype (data downloaded: November 18, 2021). Modes of inheritance were determined using OMIM-data. Genes annotated as being dominant were only retained if they were not annotated with any recessive phenotype in OMIM. The zygosity of the variants identified in the DeCOI cohort had to match the zygosity expected based on the mode of inheritance of the gene, respectively.

To reduce the risk of re-identification for the participants, identified dominant Mendelian diseases are grouped as broad categories and age ranges are reported rather than exact ages.

### Gene- and gene-set-based collapsing analyses

Next, gene- and gene-set-based collapsing analyses were conducted to study joint effects of rare variants across single genes and sets of genes with presumed importance to COVID-19. The gene- and gene-set-based collapsing analyses involved three stages.

First, the *definition of genes and gene-sets*: Variants were assigned to one of 19,630 protein-coding genes, as based on position (VEP’s annotation; column “SYMBOL”). Furthermore, five gene-sets were curated based on *a priori* evidence or biological plausibility for an involvement in COVID-19 etiology: (a) “GWAS_genes” (94 genes, closest to lead SNV and/or reported as a candidate gene at 71 risk loci identified in prior GWAS for SARS-CoV-2 related traits, including susceptibility and severity, S3 Table); (b) “IFNresponse_COVID-19_genes” (31 genes of the interferon signaling pathway, based on a recent review [30]); (c) *“*IFNresponse_reactome_genes” (185 genes of the interferon signaling pathway, based on reactome [58]); (d) “innate_db” (1,037 genes involved in the innate immunity pathway according to the InnateDB platform [59]; and (e) “Zhang_et_al” (13 genes involved in immune response to viral infection with a reported prior enrichment of LoF variants [11]). The major histocompatibility complex (MHC) region was excluded from all lists. Notably, a small overlap was present between individuals from DeCOI_EUR_ and several of the studies from which the “GWAS_genes” list was derived. However, given that this represented less than 0.04% of the entire sample used in the GWAS, the sample overlap was not expected to drive any associations.

*Second*, *the definition of functional masks for collapsing analyses*: Eleven functional masks (FM) were defined, as based on the predicted consequences of variants (see S4 Table). Briefly, coding variants were classified into categories analogous to those applied in previous studies [18,19]. These categories comprised: (a) predicted loss-of-function (pLoF) variants; and (b) four missense deleteriousness categories, as based on REVEL scores [60]. For non-coding variants, categories of promoters, 5’ and 3’ UTRs, as well as regulatory elements were defined, and CADD scores [61] were included as a proxy measure of deleteriousness. Variants located in the core regions of DNAse I hypersensitive sites (Altius index) [62] and within 1 kb to 50 kb upstream of the respective TSS were defined as variants in regulatory elements.

*Third*, *the statistical analyses*. Gene- and gene-set-based collapsing analyses were performed with regenie (version 3.1 [63]), using the DeCOI_EUR_ cohort and the generic variant set (see above). For each analysis, 11 FMs (see above) and two phenotypes (Ex, B1) were tested for association using the default additive model and the ‘—build-mask sum’ option. Based on prior evidence of varying heritability estimates for different age and sex categories [24], gene-set-analyses were also stratified for age (age lower than 60 years / greater or equal to 60 years), and for sex (male / female). For age and sex, stratification applied to both cases and controls. The covariates and options described for the GWAS were used (see section “Single-variant association analyses” below; settings “firth” and “ignore-pred”), with individual polygenic risk score (PRS) being added as a covariate (see section “Polygenic risk scoring” below). The same analysis was also run without PRS. The included variants had an MAF below 0.1%. Allele frequency was determined based on the maximum allele frequency in either the present cohort or gnomAD (version 3.1.2; all populations). Conservative Bonferroni-based thresholds for multiple corrections were alpha = 1.16x10^-07^ (19,630 genes, 11 FM, 2 phenotypes) for the single gene analyses, and alpha = 9.1x10^-05^ for the gene-set-analysis (5 sets, 11 FM, 2 phenotypes, 5 stratifications). Statistical analyses were only performed if the category contained at least one variant.

### Single-variant association analyses

For single variant analyses, two GWAS were performed in the DeCOI_EUR_ cohort using the case/control definitions Ex and B1. For each of the two GWAS, variants were removed from the generic variant list if they met any of the following criteria: MAF < 0.5%, vCR < 98%, missingness-difference between cases and controls above 2%, Hardy-Weinberg p<10^−6^ (among autosomal variants in respective controls), p<10^−10^ (among autosomal variants in cases), p<10^−6^ (among X-chromosomal variants in females). These GWAS variant sets (n = 15,708,109 variants (Ex), n = 15,742,368 (B1)) were pruned (“indep-pairwise 50 5 0.05” command, autosomal variants only, performed in PLINK, n = 548,183 (Ex) and n = 549,436 variants (B1) remaining) and used for calculation of PCs in order to capture the population structure within each GWAS. Together with age, sex, age*age, and age*sex, these 10 PCs were used as covariates in a logistic regression, which was conducted using regenie (version 3.1; options “firth” and “ignore-pred”). For the Ex case-control definition, analysis was re-run in phenotypic substrata (i.e., male/female and younger than 60 years/older or equal 60 years; see above).

### Replication cohorts/data

For selected analyses, *in silico* replication was attempted using previously generated summary statistics from the COVID-19 HGI release 7 (array-based data, without GenOMICC and 23andMe) [10] and GenOMICC (WGS data) [22]. For low frequency candidate variants, or when individual genotype data were required, WGS data from the BQC-19 project (Quebec Biobank) [64] were re-analyzed.

### Autozygosity

For each individual in the DeCOI_EUR_ cohort, the inbreeding coefficient (F_I_) was estimated in accordance with the definition proposed by Wright [65,66], and as implemented in PLINK v1.9 with the—ibc command (Fhat3). F_I_ was first calculated on the basis of all variants, and then on the basis of those with a MAF ≥ 1% (PLINK, option—maf 0.01) to evaluate the robustness of the analysis. Using the Ex and B1 case-control definitions respectively, F_I_ values between cases and controls were compared using: (i) a one-sided Wilcoxon-test; and (ii) logistic regression with 10 PCs as covariates, as described in the section “Single-variant association analyses”. The autozygosity definition follows the standard approach used by Cruz et al. [24] for their “F_GRM_” analysis. Their “F_ROH_” analysis approach, which is an ad-hoc assessment of the autozygous proportions in the human genome but not a direct autozygosity measure, was not pursued.

### Polygenic risk scoring

WGS-based GWAS data from the GenOMICC study [22], which has no known sample overlap with the DeCOI cohort, were used to generate a PRS for severe COVID-19. The program PRS-CS (version 1.0.0) [67] was applied to the summary statistics of European-ancestry individuals from GenOMICC, using the UK Biobank-based LD reference panel, as provided by PRS-CS. The resulting predictor contained 967,463 variants. PRS for individuals from the DeCOI_EUR_ cohort were then obtained using the ‘—score’ option within PLINK (version 1.9) for variants with MAF>1% of the generic variant set (required: vCR > 98%). These individual scores were included as covariates in the collapsing-analyses (described above).

P-values for the predictor PRS were determined using logistic regression (function glm within R using the parameter family = binomial(link = "logit")), which included PRS as well as the same covariates as those used in the GWAS (see above). To determine whether the PRS improved prediction, two logistic regression models were fitted: (i) with the covariates only; and (ii) with the covariates and the PRS, as described above. Subsequently, the Nakelkerke R^2^ was calculated for both models (NagelkerkeR2 function of the R package fmsb). The significance of the differences between the two models were then determined using the likelihood ratio test (lrtest function of the R package rms).

Since logistic regression models can be biased towards the sample used (overfitting), glmnet was also employed, since this provides a combination of ridge and lasso regressions, and is more suitable for the prediction on unknown data. To determine whether PRS added value over random noise, 100 predictors from a normal distribution were simulated, and these were used to train glmnet. To estimate the effect size using independent test data, multiple (1,000) subsampling of our dataset was performed using a random proportion of individuals from 75% to 95% for training, and the remaining dataset for testing. The unequal size of the training set was necessary in order to address the discrete nature of the data and the lack of variability on comparatively small samples. As a training procedure, cross-validation was used for choosing the optimal parameter, and glmnet was used for the model. Instead of an absolute optimum, lambda plus one standard error was chosen as a more conservative estimate. Statistical analyses were performed as implemented in glmnet (see URL).

## Supporting information

S1 Fig Schematic representation of the quality control (QC) process. After alignment and joint calling of SNVs and Indels, 1,275 individuals with appropriate phenotype data underwent sample quality control to yield a final dataset consisting of 1,017 unrelated individuals of European ancestry (DeCOI_EUR_).
(JPG)

S2 Fig Principal component analysis. For each individual, principal components were calculated based on the “common variants for QC” variant set. (A) The first two principal components (PC1 and PC2) are plotted for all individuals of DeCOI (empty forms) together with individuals from the 1000 genomes project (1 KG reference cohort, grey circles). Individuals assigned to the European subcohort of DeCOI (DeCOI_EUR_) are plotted in blue circles, while all others are indicated in black triangles. The region marked by the dashed box is enlarged in panels B-D. (B) and (C): The individuals of DeCOI_EUR_ are plotted within the PC-space, colored by their case-control definitions in analyses Ex and B1. In (D), all individuals of DeCOI_EUR_ are plotted with colors indicating their respective site of sequencing.
(JPG)

S3 Fig Characteristics of carriers of pathogenic variants with established links to monogenic diseases. (A) Box plot indicating the age distribution of individuals in which a heterozygous (filled with checkerboard pattern) or biallelic (blue data points, includes compound heterozygous) variant with an established link to a monogenic disease was or was not found (filled in white). The elements of the box plot correspond to the following values: thick line: median, box: 25th and 75th percentile, whiskers: largest / smallest value not further away from the box than 1.5 times the interquartile range, points: values outside of the range of the whiskers. Panels (B) to (D) show the proportion of heterozygous variant carriers according to cohort membership (B), severity (C) or sex (D). The numbers above the bars indicate the total number of individuals in each stratum. Note that statistical testing was performed using student’s t-test for age (A) or fisher’s exact test (B-D). Except for nominally significant differences in age, no statistically significant different proportions between strata were detected (lowest nominal p-value: 0.13). p_nom_: uncorrected p-value.
(JPG)

S4 Fig Gene-based collapsing analyses in DeCOI_EUR_. (A-B) Quantile-quantile plots for phenotypes Ex (A) and B1 (B). (C-D) Scatter plots showing the negative decadic logarithm of the p-values for gene / functional mask combinations when PRS was included (x-axis) or not included (y-axis) as a covariate. The p-values were calculated using the phenotype definitions, as indicated in the left upper corner of the scatter plots. Pearson correlation coefficients between negative decadic logarithms of the p-values calculated with or without PRS as covariate were 0.92 for Ex and 0.96 for both B1.
(JPG)

S5 Fig Quantile-quantile (QQ) plots of GWAS. Phenotypes and corresponding genomic inflation factors (lambda) are indicated within the respective panels.
(JPG)

S6 Fig Results of stratified analyses within Ex. Manhattan plots (left panel) and quantile-quantile plots (right panel) are represented for analyses including individuals which were of female (Ex_female) or male (Ex_male) sex, and younger than 60 years (Ex_LT60) or 60 years or older (Ex_GE60). Details on all variants with P<10^−05^ in any of the four substrata are listed in S13 Table.
(JPG)

S7 Fig Distribution of autozygosity in samples of the DeCOI_EUR_ cohort. Distribution of inbreeding coefficients in cases and controls according to the B1 and Ex classifications. The dashed horizontal lines represent thresholds of 0.02 (green), 0.05 (blue) and 0.1 (red), respectively.
(JPG)

S8 Fig Comparison of PCs in samples of DeCOI_EUR_ cohort. Values of principal component 1 and 2 for individuals of the DeCOI_EUR_ cohort are shown for different ranges of the inbreeding coefficient (FI). Case / control status for B1 (left) or Ex (right) is color coded only, if individuals were within the specified range of FI, otherwise individuals are colored in grey.
(JPG)

S1 Table Description of individual cohorts. (XLSX)

S2 Table Characteristics of the overall DeCOI cohort (left) and the European subcohort (DeCOI_EUR_, right).
(XLSX)

S3 Table Overview of genes used in five different gene-sets. (XLSX)

S4 Table Definition of functional masks for gene collapsing analyses. (XLSX)

S5 Table Rare variants within coding and non-coding regions of *TLR7*. (XLSX)

S6 Table Replication results for rs192357402 in the Quebec Biobank. (XLSX)

S7 Table Variants in 13 genes previously implicated in severe COVID-19 and characteristics of carriers. (XLSX)

S8 Table Results of gene collapsing analysis in Ex. This table contains the 5000 most significant results, for a full list please refer to the Data Availability section.
(XLSX)

S9 Table Results of gene collapsing analysis in B1. This table contains the 5000 most significant results, for a full list please refer to the Data Availability section.
(XLSX)

S10 Table Results of gene-set analyses. (XLSX)

S11 Table Results of most significant variants in B1 analysis of DeCOI_EUR_. (XLSX)

S12 Table Association results for known risk loci. (XLSX)

S13 Table Results of age- and sex-stratified single variant association analysis in B1. All variants that have P<10–05 in at least one subcategory are shown.
(XLSX)

S14 Table Mean PRS values in cases and controls. (XLSX)

S15 Table Names and affiliations of members of the DeCOI host genetics group. (XLSX)

S16 Table Names and affiliations of members of the DeCOI group. (XLSX)

S1 Text This file contains additional information and references on the four autosomal-recessive genes. (PDF)
