# Supplementary figures and images for: Systematic assessment of COVID-19 host genetics using whole genome sequencing data
Source: PLoS Pathog. 2024 Dec 23;20(12):e1012786. doi: 10.1371/journal.ppat.1012786 (PMC11706450; doi:10.1371/journal.ppat.1012786)

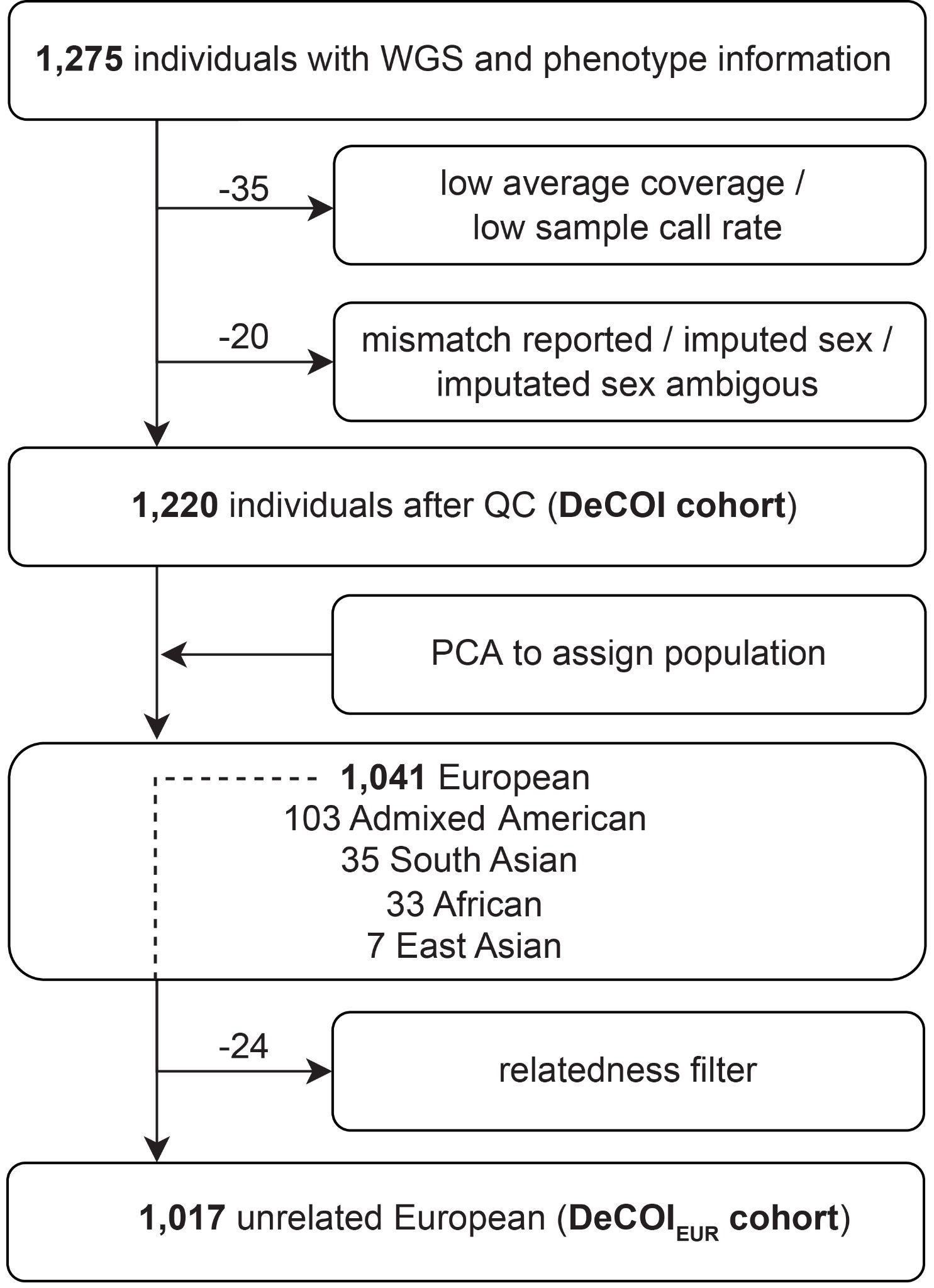

Supplement: S1 Fig — After alignment and joint calling of SNVs and Indels, 1,275 individuals with appropriate phenotype data underwent sample quality control to yield a final dataset consisting of 1,017 unrelated individuals of European ancestry (DeCOIEUR). (JPG) [file ppat.1012786.s001.jpg]

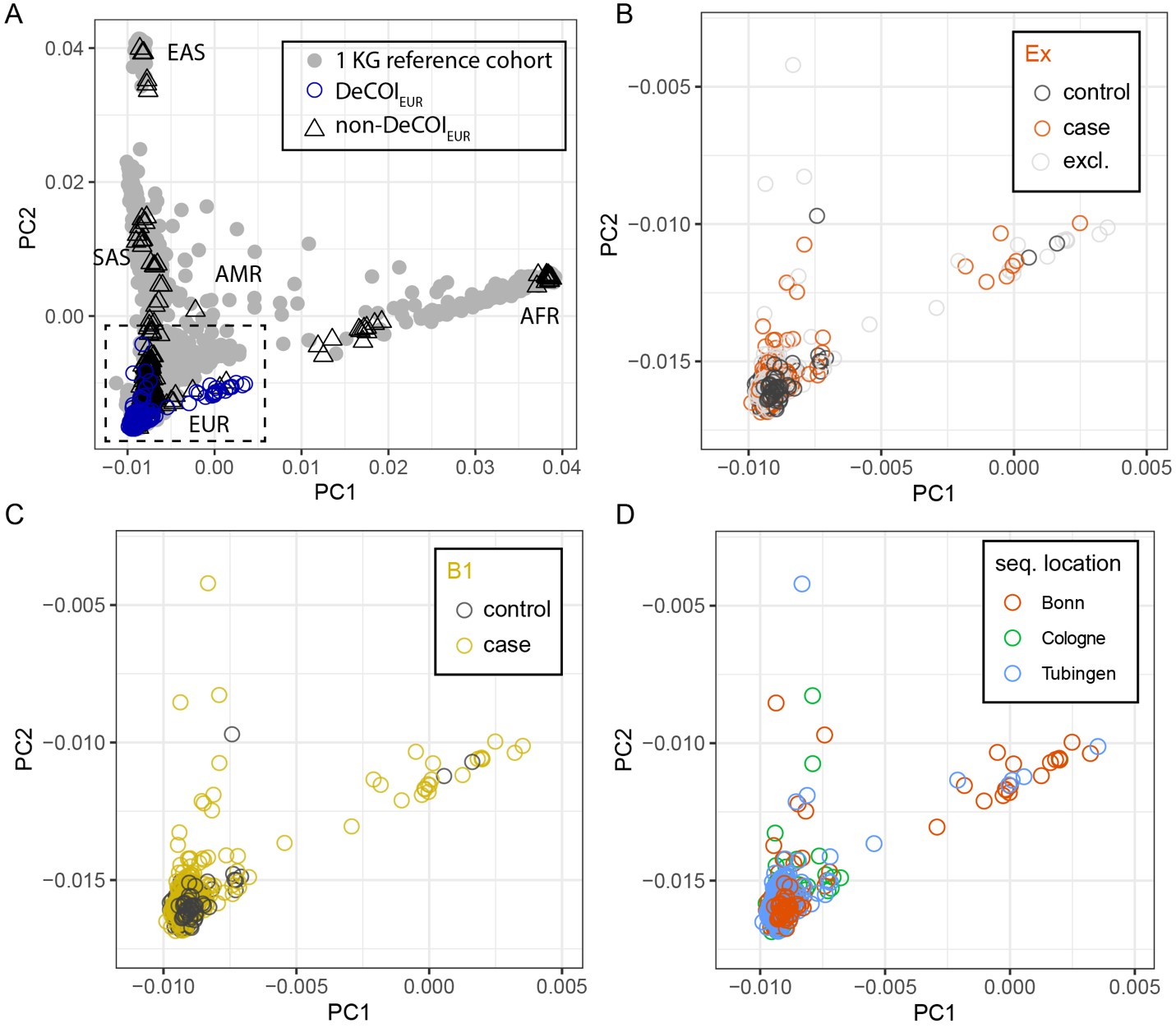

Supplement: S2 Fig — For each individual, principal components were calculated based on the “common variants for QC” variant set. (A) The first two principal components (PC1 and PC2) are plotted for all individuals of DeCOI (empty forms) together with individuals from the 1000 genomes project (1 KG reference cohort, grey circles). Individuals assigned to the European subcohort of DeCOI (DeCOIEUR) are plotted in blue circles, while all others are indicated in black triangles. The region marked by the dashed box is enlarged in panels B-D. (B) and (C): The individuals of DeCOIEUR are plotted within the PC-space, colored by their case-control definitions in analyses Ex and B1. In (D), all individuals of DeCOIEUR are plotted with colors indicating their respective site of sequencing. (JPG) [file ppat.1012786.s002.jpg]

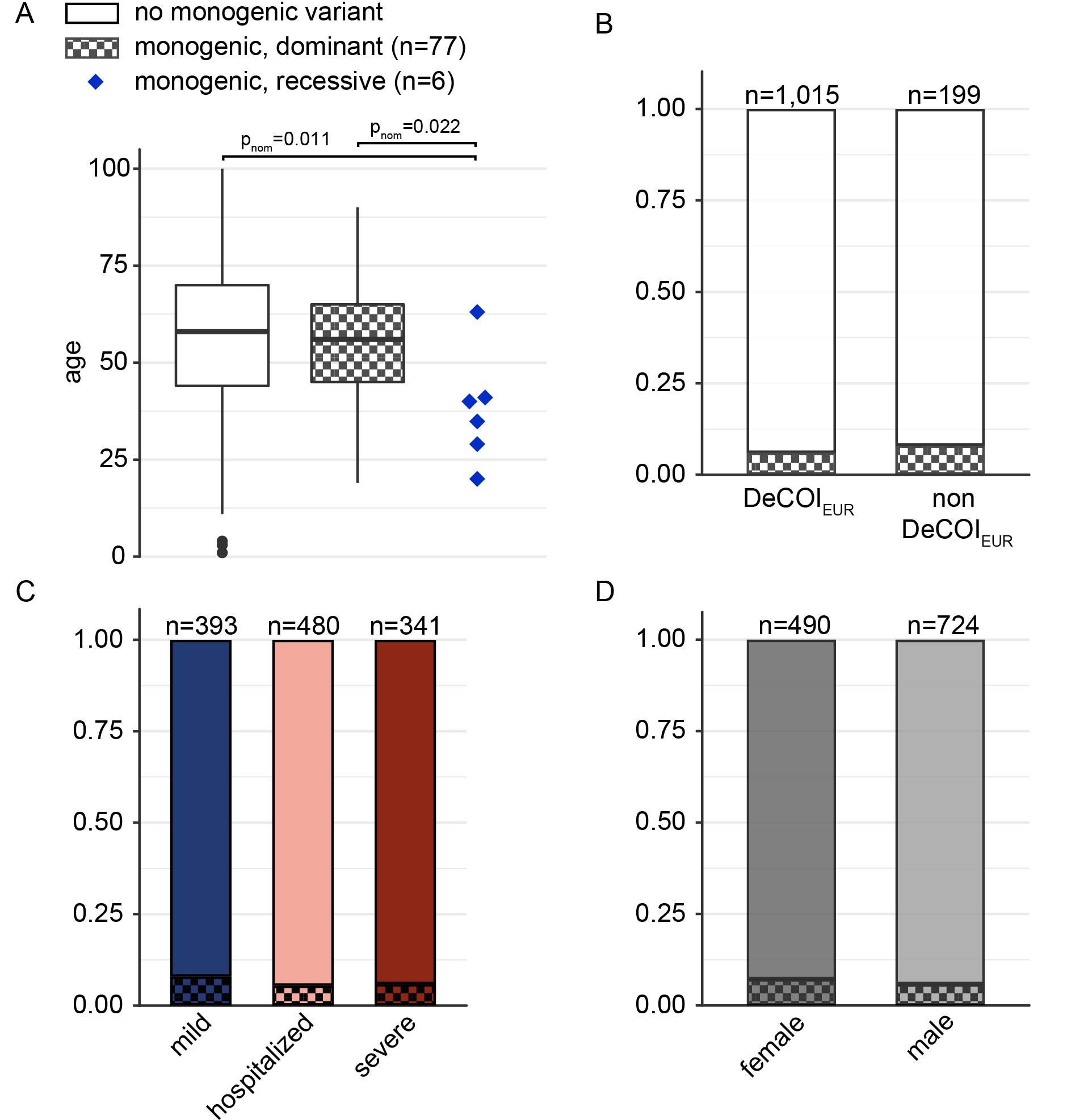

Supplement: S3 Fig — (A) Box plot indicating the age distribution of individuals in which a heterozygous (filled with checkerboard pattern) or biallelic (blue data points, includes compound heterozygous) variant with an established link to a monogenic disease was or was not found (filled in white). The elements of the box plot correspond to the following values: thick line: median, box: 25th and 75th percentile, whiskers: largest / smallest value not further away from the box than 1.5 times the interquartile range, points: values outside of the range of the whiskers. Panels (B) to (D) show the proportion of heterozygous variant carriers according to cohort membership (B), severity (C) or sex (D). The numbers above the bars indicate the total number of individuals in each stratum. Note that statistical testing was performed using student’s t-test for age (A) or fisher’s exact test (B-D). Except for nominally significant differences in age, no statistically significant different proportions between strata were detected (lowest nominal p-value: 0.13). pnom: uncorrected p-value. (JPG) [file ppat.1012786.s003.jpg]

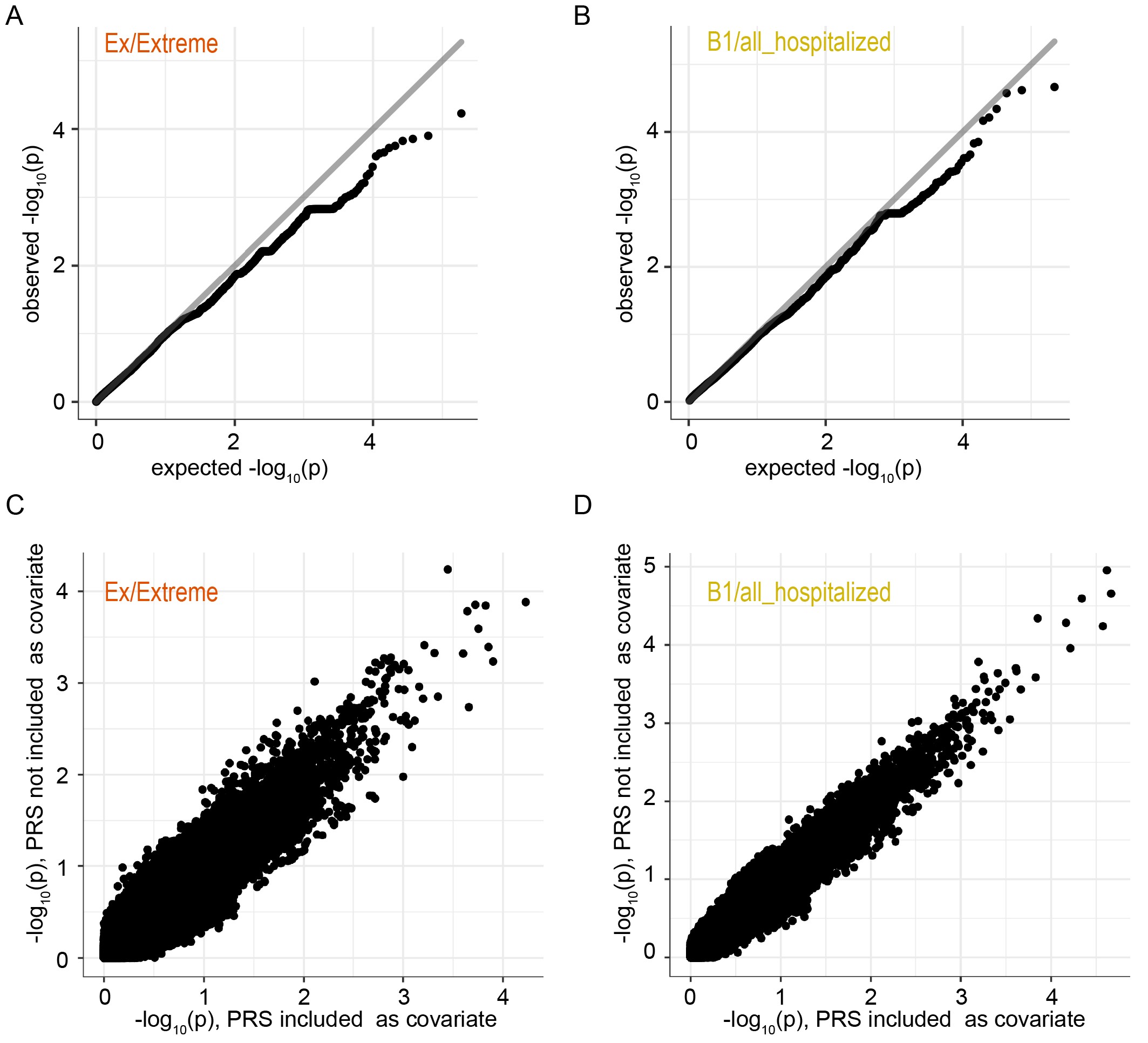

Supplement: S4 Fig — (A-B) Quantile-quantile plots for phenotypes Ex (A) and B1 (B). (C-D) Scatter plots showing the negative decadic logarithm of the p-values for gene / functional mask combinations when PRS was included (x-axis) or not included (y-axis) as a covariate. The p-values were calculated using the phenotype definitions, as indicated in the left upper corner of the scatter plots. Pearson correlation coefficients between negative decadic logarithms of the p-values calculated with or without PRS as covariate were 0.92 for Ex and 0.96 for both B1. (JPG) [file ppat.1012786.s004.jpg]

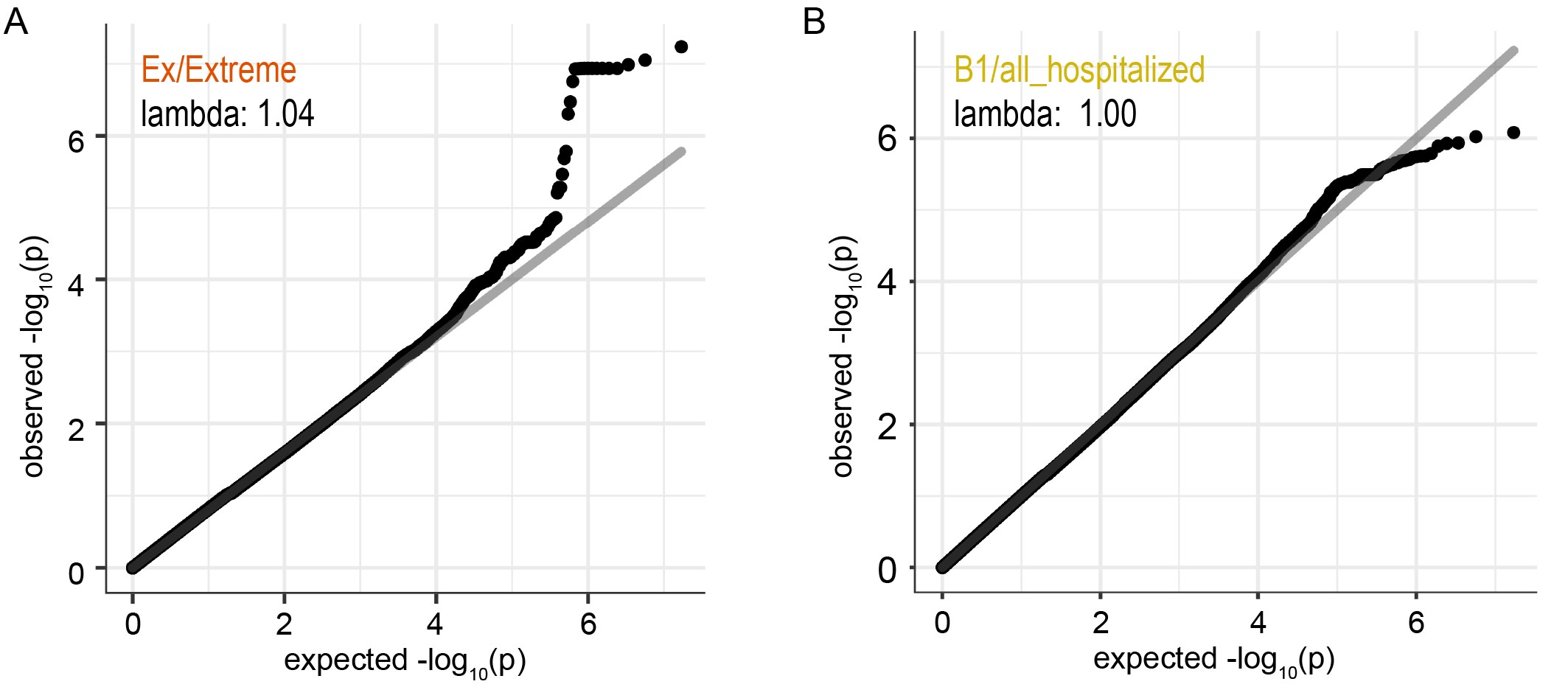

Supplement: S5 Fig — Phenotypes and corresponding genomic inflation factors (lambda) are indicated within the respective panels. (JPG) [file ppat.1012786.s005.jpg]

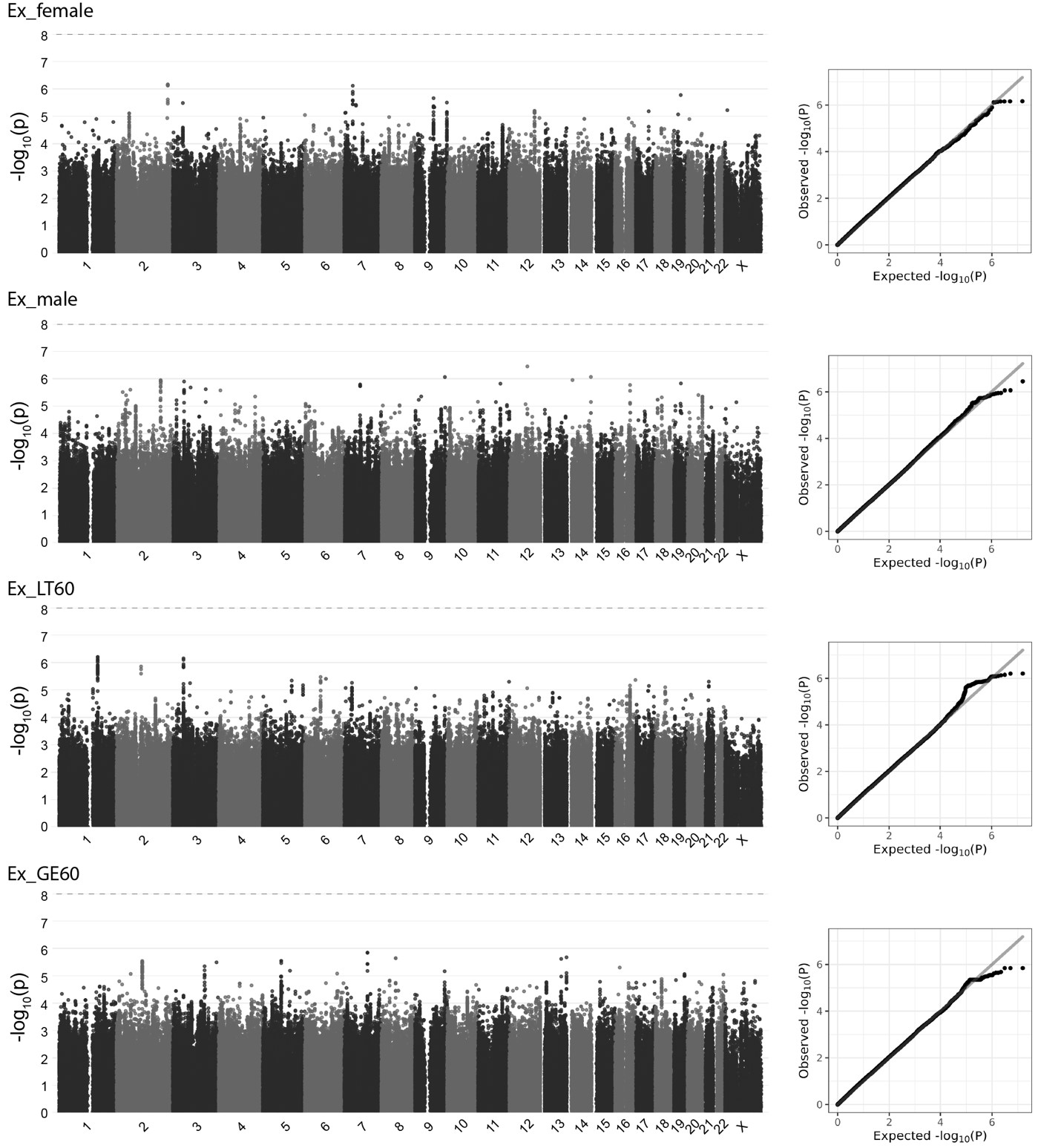

Supplement: S6 Fig — Manhattan plots (left panel) and quantile-quantile plots (right panel) are represented for analyses including individuals which were of female (Ex_female) or male (Ex_male) sex, and younger than 60 years (Ex_LT60) or 60 years or older (Ex_GE60). Details on all variants with P<10−05 in any of the four substrata are listed in S13 Table. (JPG) [file ppat.1012786.s006.jpg]

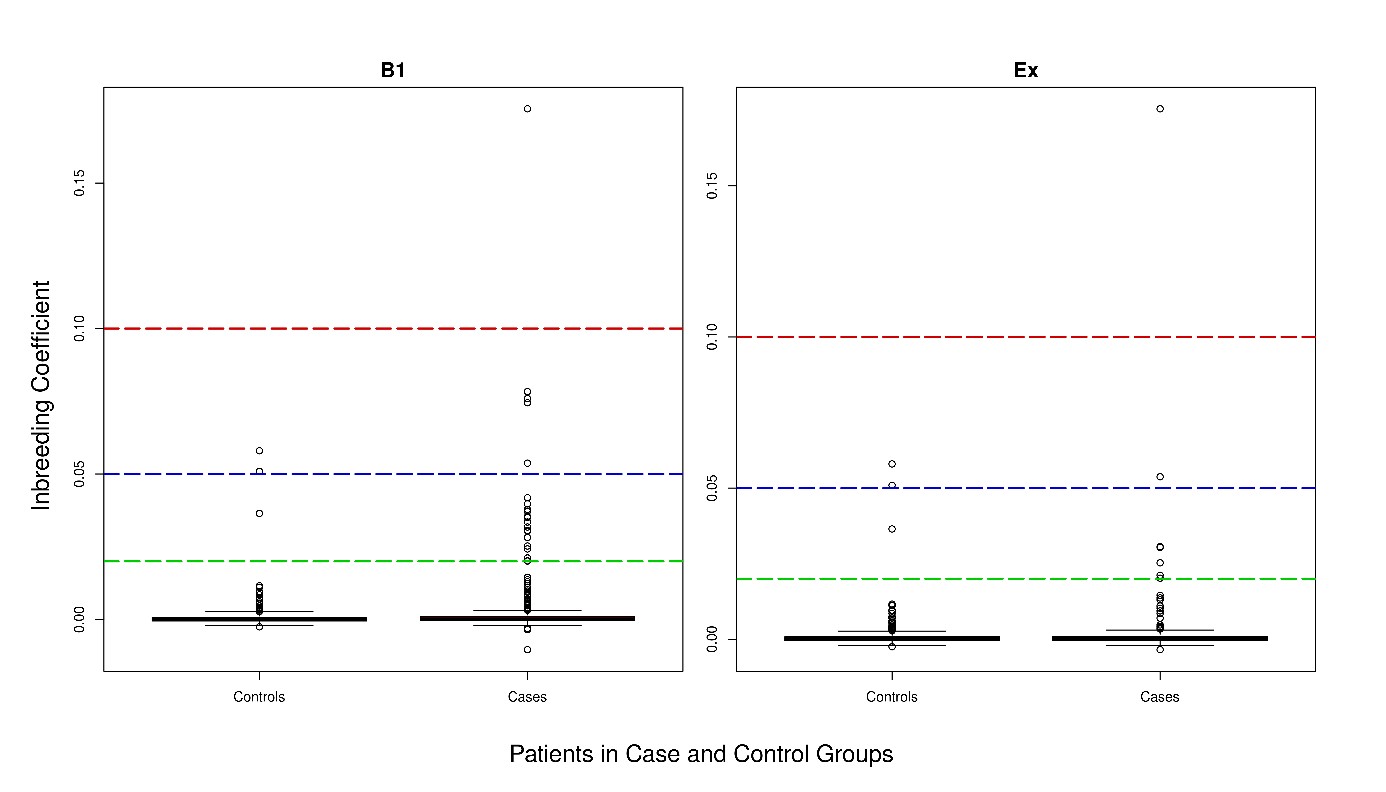

Supplement: S7 Fig — Distribution of inbreeding coefficients in cases and controls according to the B1 and Ex classifications. The dashed horizontal lines represent thresholds of 0.02 (green), 0.05 (blue) and 0.1 (red), respectively. (JPG) [file ppat.1012786.s007.jpg]
