# Supplementary material for: Systematic assessment of COVID-19 host genetics using whole genome sequencing data
Source: PLoS Pathog. 2024 Dec 23;20(12):e1012786. doi: 10.1371/journal.ppat.1012786 (PMC11706450; doi:10.1371/journal.ppat.1012786)
Supplement: S1 Text — (PDF) [file ppat.1012786.s025.pdf]

Supplementary Text

In addition to *AIRE*, whose relevance in the context of COVID-19 has been described in the main text, we here provide information on the remaining 5 genes underlying monogenic autosomal recessive disorders:

BBS1. The present analyses identified a homozygous pathogenic variant in *BBS1* in an individual with lethal COVID-19. This *BBS1* variant causes the ciliopathy Bardet-Biedl Syndrome 1, which is characterized by variable manifestations including polydactyly, obesity, retinal disease, cognitive impairment, and abnormalities of the urogenital system. While respiratory symptoms are not typical in Bardet-Biedl syndrome, alterations in the function of the respiratory epithelium, and increased rates of both neonatal respiratory distress and asthma, have been reported (1–3), which might render an individual with Bardet-Biedl syndrome more susceptible to a severe COVID-19 disease course. Consistent with Bardet-Biedl syndrome, the individual from our cohort was reported to have an intellectual developmental disorder. Epidemiological data suggest that intellectual developmental disorders may contribute to the risk for severe COVID-19 (4).

AGXT. We identified a pathogenic variant present in homozygous state in *AGXT,* which is causing primary hyperoxaluria according to ClinVar. In our study the variant was identified in a young man with lethal COVID-19, who had undergone kidney and liver transplantation. While it is plausible that both the pre-existing organ damage secondary to oxalosis, and the treatment of the disease (renal and liver transplantation and immunosuppression), had a negative influence on COVID-19 disease course, no additional data were available with which to differentiate between these two possibilities.

SERPIN1C. A pathogenic homozygous variant linked to antithrombin III deficiency was identified in a moderately affected male. Antithrombin III deficiency could augment existing thrombophilic tendencies in COVID-19, or interfere with standard antithrombotic pharmacological management strategies (5,6).

HBB. One individual with likely compound heterozygous beta-thalassemia major had moderate COVID-19. For individuals with beta-thalassemia a lack of increased risk of contracting SARS-CoV-2 infection or severe COVID-19 was reported (7,8).

PAH. In another patient, likely compound heterozygous pathogenic variants were observed in *PAH*, which are associated with mild Hyperphenylalaninemia and mild Phenylketonuria (PKU). The severity of COVID-19 has been shown to be significantly associated with higher levels of phenylalanine (9).

References (Supplementary Text)

1. Viehl L, Wegner DJ, Hmiel SP, White FV, Jain S, Cole FS, et al. Lethal neonatal respiratory failure due to biallelic variants in BBS1 and monoallelic variant in TTC21B. Pediatr Nephrol Berl Ger. 2023 Feb;38(2):605–9.

2. Shah AS, Farmen SL, Moninger TO, Businga TR, Andrews MP, Bugge K, et al. Loss of Bardet-Biedl syndrome proteins alters the morphology and function of motile cilia in airway epithelia. Proc Natl Acad Sci U S A. 2008 Mar 4;105(9):3380–5.

3. Shoemark A, Dixon M, Beales PL, Hogg CL. Bardet Biedl syndrome: motile ciliary phenotype. Chest. 2015 Mar;147(3):764–70.

4. Koks-Leensen MCJ, Schalk BWM, Bakker-van Gijssel EJ, Timen A, Nägele ME, van den Bemd M, et al. Risk for Severe COVID-19 Outcomes among Persons with Intellectual Disabilities, the Netherlands. Emerg Infect Dis. 2023 Jan;29(1):118–26.

5. de la Morena-Barrio ME, Gindele R, Bravo-Pérez C, Ilonczai P, Zuazu I, Speker M, et al. High penetrance of inferior vena cava system atresia in severe thrombophilia caused by homozygous antithrombin Budapest 3 variant: Description of a new syndrome. Am J Hematol. 2021 Nov 1;96(11):1363–73.

6. Abou-Ismail MY, Diamond A, Kapoor S, Arafah Y, Nayak L. The hypercoagulable state in COVID-19: Incidence, pathophysiology, and management. Thromb Res. 2020 Oct;194:101–15.

7. Vacca N, Locci C, Serra F, Chicconi E, Puci MV, Sotgiu G, et al. Impact of COVID-19 on patients with beta-thalassemia major: an observational study. Acta Haematol. 2024 Feb 28;

8. Ghoti H, Zreid H, Ghoti I, Bourgonje AR, Diepstra A, van Goor H, et al. Clinical outcome and humoral immune responses of β-thalassemia major patients with severe iron overload to SARS-CoV-2 infection and vaccination: a prospective cohort study. EClinicalMedicine. 2023 Aug;62:102096.

9. Luporini RL, Pott-Junior H, Di Medeiros Leal MCB, Castro A, Ferreira AG, Cominetti MR, et al. Phenylalanine and COVID-19: Tracking disease severity markers. Int Immunopharmacol. 2021 Dec;101(Pt A):108313.
